# NUDT16 regulates CtIP PARylation to dictate homologous recombination repair

**DOI:** 10.1093/nar/gkae064

**Published:** 2024-02-07

**Authors:** Zhen Zhang, William E Samsa, Zihua Gong

**Affiliations:** Department of Cancer Biology, Cleveland Clinic Lerner Research Institute, Cleveland, OH, USA; Department of Cancer Biology, Cleveland Clinic Lerner Research Institute, Cleveland, OH, USA; Department of Cancer Biology, Cleveland Clinic Lerner Research Institute, Cleveland, OH, USA

## Abstract

CtIP initiates DNA end resection and mediates homologous recombination (HR) repair. However, the underlying mechanisms of CtIP regulation and how the control of its regulation affects DNA repair remain incompletely characterized. In this study, NUDT16 loss decreases CtIP protein levels and impairs CtIP recruitment to double-strand breaks (DSBs). Furthermore, overexpression of a catalytically inactive NUDT16 mutant is unable to rescue decreased CtIP protein and impaired CtIP recruitment to DSBs. In addition, we identified a novel posttranslational modification of CtIP by ADP-ribosylation that is targeted by a PAR-binding E3 ubiquitin ligase, RNF146, leading to CtIP ubiquitination and degradation. These data suggest that the hydrolase activity of NUDT16 plays a major role in controlling CtIP protein levels. Notably, ADP-ribosylation of CtIP is required for its interaction with NUDT16, its localization at DSBs, and for HR repair. Interestingly, NUDT16 can also be ADP-ribosylated. The ADP-ribosylated NUDT16 is critical for CtIP protein stability, CtIP recruitment to DSBs, and HR repair in response to DNA damage. In summary, we demonstrate that NUDT16 and its PARylation regulate CtIP stability and CtIP recruitment to DSBs, providing new insights into our understanding of the regulation of CtIP-mediated DNA end resection in the HR repair pathway.

## Graphical Abstract

**Graphical Abstract:**
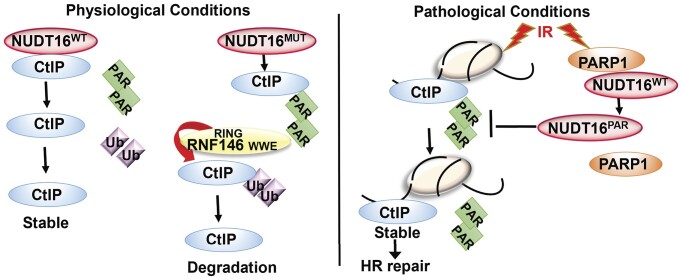

## Introduction

DNA double-strand breaks (DSBs) are highly toxic DNA lesions and require timely repair by dedicated machinery, because one unrepaired DSB is sufficient to trigger permanent growth arrest and cell death. Two mechanistically distinct pathways have evolved to eliminate DSBs from the genome: non-homologous end joining (NHEJ), which rejoins the broken ends of a severed DNA molecule, and homologous recombination (HR), which requires large stretches of identical or very similar DNA sequences elsewhere in the genome to serve as templates for DNA repair (1–3). DSB repair regulation is of great importance to human health because errors in selecting the appropriate DSB repair pathway can drive cancer development. The current hypothesis is that BRCA1- and 53BP1-dependent pathways are competing during the early stages of HR repair, particularly at DNA end resection. End resection is the key step in determining repair pathway choice, since ends that bear long 3′ DNA tails are destined for HR repair, while at the same time such ends are not substrates for NHEJ and therefore would prevent NHEJ repair. While BRCA1 promotes DNA end resection, 53BP1 may suppress end resection and therefore promote NHEJ and prevent HR repair (4–8).

DNA end resection plays a key role in HR repair. DNA end resection is initiated by the CtBP-interacting protein (CtIP) together with the MRE11–RAD50–NBS1 (MRN) complex to generate a short ssDNA at the DSB ends. Subsequently, downstream nucleases exonuclease 1 (EXO1), DNA replication helicase/nuclease 2 (DNA2), or Bloom syndrome protein (BLM) extends the 3′-ssDNA (9). The ssDNAs are rapidly coated by the ssDNA-binding protein RPA. The displacement of RPA from ssDNA results in the formation of the RAD51-nucleoprotein filament, which in turn catalyzes strand invasion and homology search (10). CtIP is required for DNA end resection and therefore is necessary for HR (11). Interestingly, CtIP can also participate in DSB repair by microhomology-mediate end-joining (MMEJ) during the G1 phase (12). CtIP physically interacts with the NBS1 subunit of the MRN complex (13). Given that CtIP recruitment requires ATM kinase activity, and that ATM activation depends on the MRN complex, it is plausible that the MRN complex plays an indirect role in the initial recruitment of CtIP by activating ATM (11).

The NUDIX-type hydrolase superfamily hydrolyzes a wide range of substrates, thereby playing an essential role in important biological processes, such as cell proliferation, signal transduction and homeostasis (14–16). NUDT16 is a member of the Nudix hydrolase family and catabolizes nucleoside triphosphates and capped mRNAs, and possesses important DNA protective roles (17,18). NUDT16 has been shown to have *in vitro* hydrolase activities that remove protein ADP-ribosylation (19,20). Our recent work demonstrated that NUDT16 cleaves the ADP-ribosylation of 53BP1 and inhibits 53BP1 ubiquitination and degradation, thus stabilizing 53BP1 protein (20). In this study, we investigated whether NUDT16 regulates other DNA repair proteins, and we are now reporting that by hydrolyzing poly (ADP-Ribosyl)ation (PAR) chains on CtIP, NUDT16 regulates CtIP stability and further dictates the end resection-initiated HR repair pathway in mammalian cells.

## Materials and methods

### Cell culture and plasmids

HEK293T, MCF10A, MDA-MB-231 and U2OS cells were purchased from American Type Culture Collection (ATCC) and cultured under conditions specified by the manufacturer. EJ2-GFP U2OS cell, EJ5-GFP U2OS cell, EJ7-GFP-U2OS cell and DR-GFP-U2OS cell were obtained from Dr Jeremy Stark, Beckman Research Institute of the City of Hope, Duarte, CA. The DIvA cell line was obtained from Dr Gaelle Legube, CNRS-University of Toulouse, France. NUDT16 and CtIP cDNA was subcloned into pDONR201 as an entry vector and subsequently transferred to gateway-compatible destination vectors for the expression of the triple-epitope tag SFB (S protein, FLAG, and streptavidin-binding peptide), Myc and MBP epitope fusion proteins. All deletion mutants were generated by site-directed mutagenesis and verified by DNA sequencing.

### Antibodies

The NUDT16 antibody was purchased from Proteintech Group. The anti-phospho-RPA2 (S4/S8) was obtained from Bethyl Laboratories. The anti-FLAG M2 antibody was purchased from Sigma. Anti-PARP1 and anti-GAPDH antibodies were obtained from Santa Cruz. Anti-PAR and anti-PARP1 antibodies were purchased from Trevigen. The anti-RPA32 and anti-ubiquitin antibodies were purchased from Abcam. The anti-KU70, anti-KU80, anti-Myc and anti-α-Tubulin antibodies were obtained from Cell Signaling Technology. The anti-CtIP antibody was purchased from Active motif. The anti-MBP antibody was purchased from BioLegend.

### Immunofluorescence staining

Cells grown on coverslips were mock-treated or irradiated with a JL Shepherd Cs137 and allowed to recover for different time points. Cells were pre-extracted for 5 min with CSK buffer (100 mM NaCl, 300 mM sucrose, 10 mM Pipes, pH 6.8, 3 mM MgCl_2_) with 0.5% Triton X-100, and then fixed in 4% paraformaldehyde solution for 10 min. Cells were incubated with primary antibodies diluted in 5% goat serum at room temperature for 2 h. Coverslips were washed and incubated with secondary antibodies for 1 h at room temperature. Cells were then stained with DAPI to visualize nuclear DNA. The coverslips were mounted onto glass slides with anti-fade solution and visualized using the Nikon Eclipse E800 fluorescence microscope.

### Co-immunoprecipitation and western blotting

Cells were lysed with NTEN buffer (20 mM Tris–HCl, pH 8.0, 100 mM NaCl, 1 mM EDTA, 0.5% Nonidet *P*-40) containing protease inhibitors and phosphatase inhibitors at 4°C for 30 min. Cleared cell lysates were incubated with either Protein A/G agarose bead coupled with anti-NUDT16, anti-CtIP, anti-PARP1 and anti-PAR antibodies or streptavidin Sepharose beads for 3 h at 4°C. Beads were then washed and boiled in 2× Laemmli buffer and separated by SDS-PAGE. PVDF membranes were blocked in 5% milk in TBST buffer and then probed with antibodies as indicated.

### Recombinant protein production

The MBP fusion proteins expressed in *Escherichia coli* were purified by amylose resin and eluted by maltose buffer (10 mM maltose, 20 mM Tris–HCl, pH 7.4, 200 mM NaCl, 1 mM EDTA, 10 mM β-mercaptoethanol).

### Pull-down assays using bacterially expressed fusion proteins

MBP fusion proteins were expressed in *E. coli* and purified. MBP fusion proteins were immobilized on amylose resin and incubated with lysates prepared from cells transiently transfected with plasmids encoding the indicated proteins. The resulting samples were subjected to SDS-PAGE and analyzed by western blotting.

### *In vitro* PARylation assay

HEK293T cells were transfected with plasmids encoding SFB-tagged proteins. Cells were lysed with NTEN buffer containing protease inhibitors and phosphatase inhibitors on ice for 30 min. Clear cell lysates were incubated with streptavidin beads at 4°C for 3 h. Then the beads were eluted with 2 mg/ml biotin in NTEN buffer containing protease inhibitors and phosphatase inhibitors at 4°C for 2 h. *In vitro* PARylation assays were performed in a reaction mix consisting of the eluates, 1× reaction buffer (0.5 M Tris/HCl pH 8.0, 0.04M MgCl_2_, 0.5M NaCl, 2 mM DTT), 20 μM NAD+ and 1× active DNA for 30 min at room temperature. The reaction mixture was terminated with 2× Laemmli buffer and subjected to SDS–PAGE. PARP1 enzyme in a reaction mix without eluates serves as a positive control.

### *In situ* proximity ligation assay (PLA)

U2OS cells were cultured on coverslips, fixed with 4% paraformaldehyde for 10 min and permeabilized with 0.5% Triton X-100 for 5 min, followed by 1 h blocking in blocking buffer. For the visualization of protein interactions, samples were incubated with the primary antibodies for 1 h at room temperature. *In situ* PLA was performed according to the manufacturer's protocol (Duolink® Proximity Ligation Assay, Sigma) using PLA probe anti-mouse MINUS and PLA probe anti-rabbit PLUS.

### siRNA mediated knockdown

All siRNA oligonucleotides used in this study, NUDT16 siRNA (#s43640, #s43642), CtIP siRNA (#s11849, #s11850) and control siRNA (#4390844), were synthesized from Thermo Fisher Scientific. siRNAs targeting NUDT16 or CtIP were transfected to DR-GFP-U2OS, EJ5-GFP-U2OS, EJ2-GFP-U2OS, EJ7-GFP-U2OS or DIvA cells using Lipofectamine 3000 (Life Technologies) according to the manufacturer's instructions. The knockdown efficiency was confirmed by western blot analysis.

### DSB repair reporter assays

**Figure 1.**
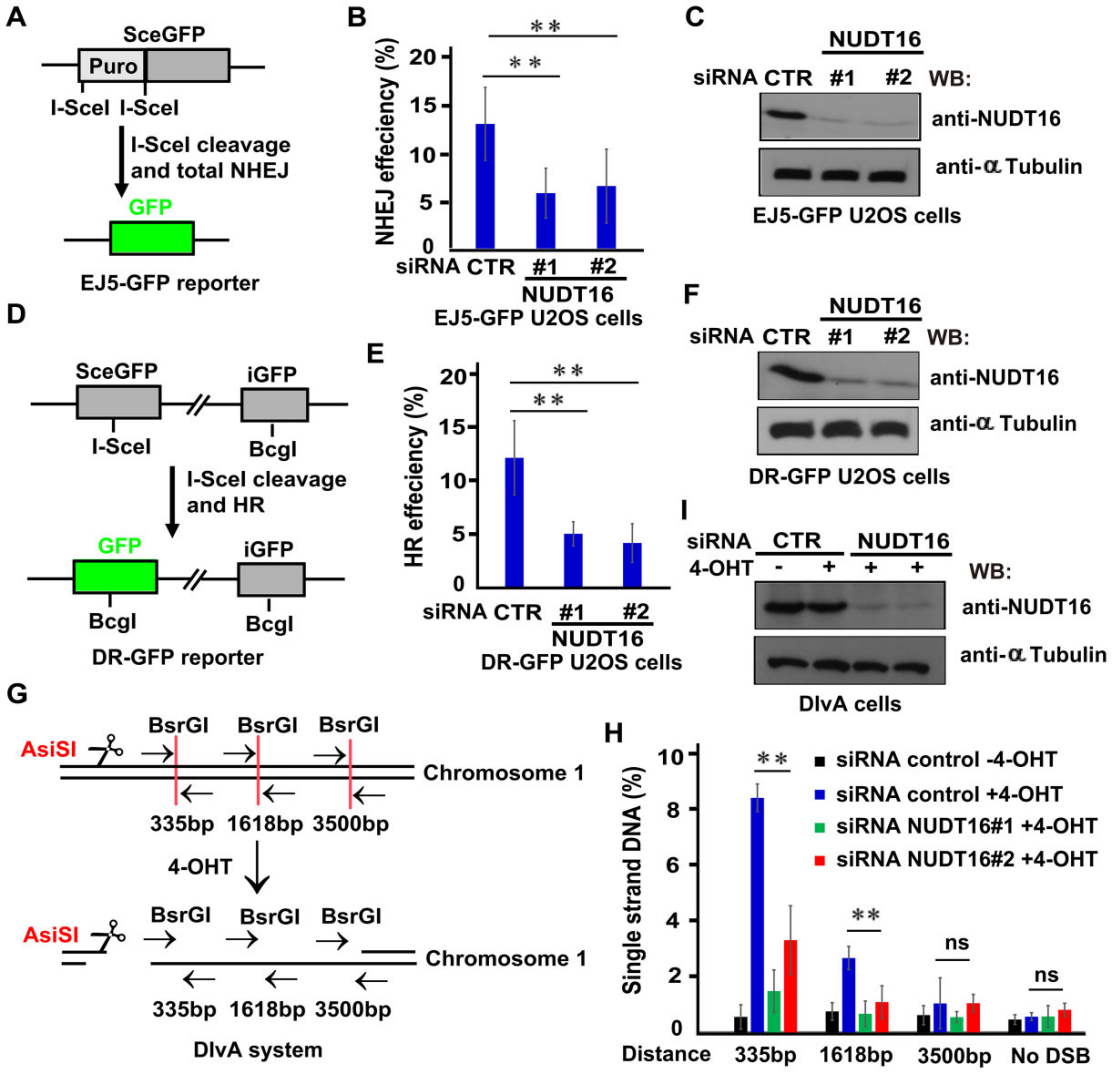
NUDT16 is involved in the DNA repair pathway. (**A–C**) NUDT16 participates in NHEJ repair. (**A**) Schematic representation of the EJ5-GFP reporter used to analyze the repair of *I-SceI*-induced DSBs by total NHEJ. (**B**) 24 h after transfection with NUDT16 siRNAs, EJ5-GFP U2OS cells were transfected with an I-SceI plasmid and pcDNA3.1-mCherry at a 9:1 ratio for 48 h. The GFP^+^ and mCherry^+^ population was quantitated and the NHEJ rate represented as GFP^+^/mCherry^+^. Data are represented as the mean ± S.E. (*n*= 3). **P< 0.01. (**C**) Western blotting analysis was performed to verify efficient silencing of NUDT16 following siRNA transfection in EJ5-GFP U2OS cells. (D–F) NUDT16 participates in HR repair. (**D**) Schematic representation of the DR-GFP reporter used to analyze the repair of *I-SceI*-induced DSBs by HR. (**E**) 24 h after transfection with NUDT16 siRNA, DR-GFP U2OS cells were transfected with an I-SceI plasmid and pcDNA3.1-mCherry at a 9:1 ratio for 48 h. The GFP^+^ and mCherry^+^ population was quantitated, and the HR rate represented as GFP^+^/mCherry^+^. Data are represented as the mean ± S.E. (*n*= 3). **P< 0.01. (**F**) Western blotting analysis was performed to verify efficient silencing of NUDT16 following siRNAs transfection in DR-GFP U2OS cells. (**G**) A schematic representation for studying the quantitative DNA resection assay based on the DIvA system. (**H**) Quantitative measurement of ssDNA generation by 5′ end resection at *AsiSI*-induced DSBs. DIvA cells transfected with the indicated siRNAs were incubated with 4-OHT for 4 h or mocked treated. Genomic DNA was extracted and digested with *BsrGI* or *HindIII*. The percentage of ssDNA intermediates at indicated sites was measured by qPCR as described in the *Methods*. Data represents mean ± S.E. (*n*= 3). **P< 0.01; ns, not significant. (**I**) Western blotting analysis was performed to verify efficient silencing of NUDT16 following siRNA transfection in DIvA cells.

The well-established EJ5-GFP-U2OS, DR-GFP-U2OS, EJ7-GFP-U2OS and EJ2-GFP-U2OS reporter cell lines were used to measure total NHEJ, HR, classical NHEJ (c-NHEJ) and Alternative end-joining (Alt-EJ), respectively. In the EJ5-GFP reporter, a green fluorescent protein (GFP) cassette is separated by a Puromycin marker that is flanked by two tandem *I-SceI* sites, such that NHEJ repair that uses distal ends of the two I-SceI-induced DSBs restores GFP expression (Figure 1A). DR-GFP is composed of two differentially mutated GFP genes oriented as direct repeats: the upstream repeat contains the recognition site for the *I-SceI* endonuclease, and the downstream repeat is a 5′ and 3′ truncated GFP fragment. Transient expression of I-SceI leads to a DSB in the upstream GFP gene; HR to repair the DSB results in GFP+ cells (Figure 1D). In the EJ7-GFP reporter, the GFP coding sequence at the GGC codon (Gly 67) is split by inserting a 46-nucleotide spacer between the first two bases (GG) and the final base (C). Two sgRNAs, 7a and 7b, target Cas9-induced DSBs to excise the 46-nt spacer. The end-joining (EJ) between the distal DSBs without indels restores the GGC codon (Supplemental Figure S1E). In the EJ2-GFP reporter, the reading frame of a GFP cassette with an N-terminal tag is disrupted by an *I-SceI* site followed by stop codons in all frames, which is flanked by eight nucleotides of microhomology (Supplemental Figure S1I). Alt-EJ of an *I-SceI* generated chromosomal DSB that deletes the stop codons, which occurs predominantly via the use of 8 nucleotides of flanking microhomology to bridge the DSB, restores the GFP cassette and causes a 35-nucleotide deletion. In brief, four reporter cell lines were transfected with control, NUDT16 or CtIP siRNAs for 24 h, then transfected with I-SceI vector and pcDNA3.1-mCherry at 9:1 ratio for another 48 h. The GFP^+^ and mCherry^+^ cell population was quantitated and the total NHEJ, HR, c-NHEJ or Alt-EJ repair rate represented as GFP^+^/mCherry^+^.

### DNA end resection assay

The DIvA (DSB-induced via *AsiSI*) cell line allows the induction of DSBs at *AsiSI*-target sequence across the human genome. Pretreatment of DIvA (*AsiSI*-ER-U2OS) cells with 4-hydroxytamoxifen (4-OHT) triggers the nuclear translocation of the *AsiSI* endonuclease and results in roughly a hundred site-specific DSBs (Figure 1G). Briefly, DIvA cells were treated with 600nM 4-OHT for 4 h or mock-treated. Genomic DNA was extracted. The genomic DNA sample (∼400 ng) was subjected to an RNase H treatment for 15 min before mock digestion or digestion with the restriction enzyme *BsrGI* (DSB, chromosome 1) or *HindIII* (No DSB, chromosome 22) at 37°C overnight. Samples were heat-inactivated at 65°C for 10 min and were used as templates for qPCR. To quantify the extent of resection, the digested or mock-digested samples were amplified by qPCR using primers that are previously described (21). The percentage of ssDNA (ssDNA%) generated by resection at selected sites was calculated based on the following equation: ssDNA % = 1/(2 ^(ΔCt – 1)^ + 0.5) × 100. ΔCt was calculated by subtracting the Ct value of the mock-digested sample from the Ct value of the digested sample. At least three biological repeats were performed.

### Tandem affinity purification

293T cells stably expressing the SFB-NUDT16 were collected and lysed with NTEN buffer containing protease inhibitors and phosphatase inhibitors on ice for 20 min. Crude lysates were collected by centrifugation, and the pellets were suspended in nuclease buffer (10 mM HEPES, pH 7.4, 10 mM KCl, 0.5 mM MgCl_2_, 2 mM CaCl_2_ and 1 μg/ml of each of pepstatin A and aprotinin) supplemented with 150 U/ml micrococcal nuclease S7 and incubated in a 37°C water bath for 5 min until the suspension turned cloudy. Then the chromatin fraction was collected by centrifugation, and the supernatants and crude lysates were incubated with streptavidin-conjugated beads for 3 h at 4°C. The immunocomplexes were washed three times with the NTEN buffer and then bead-bound proteins were eluted twice with NTEN buffer containing 1 mg/ml biotin. The eluates were incubated with S protein beads. The immunocomplexes were again washed three times with NTEN buffer and subjected to SDS-PAGE. Protein bands were excised, digested and the peptides were analyzed by mass spectrometry (performed by the Proteomics and Metabolomics Core, Cleveland Clinic).

### Statistical analysis

The reported values are the means and SD of three independent experiments. Statistical analysis was performed using the Student's *t* test and one-way ANOVA test. *P*< 0.05 was considered as statistically significant.

## Results

### NUDT16 is involved in the DNA repair pathway.

Our previous work demonstrated that NUDT16 regulates 53BP1 stability and 53BP1 recruitment to DSBs in response to DNA damage (20). To explore the functional significance of NUDT16 in DSB repair, we analyzed the activity of the two DSB repair pathways, NHEJ and HR, using the previously descried EJ5-GFP and DR-GFP reporter systems (22,23), respectively. In general, the DSB repair reporter cells harbor a disrupted GFP gene, and expression of intact GFP requires successful repair of an *I-SceI* or Cas9–induced DSB at the gene locus and can be quantified. SiRNA mediated knockdown of NUDT16 in EJ5-GFP-U2OS cells caused a significant reduction of the GFP-positive cells compared with the control cells (Figure 1A–C, Supplemental Figure S1A). Moreover, NUDT16 silencing decreased HR repair activity when assessed using the DR-GFP system (Figure 1D–F, Supplemental Figure S1B). DNA end resection is a key step in HR-mediated DNA DSB repair (24). To this end, we performed a quantitative DNA resection assay based on the DIvA system (25). Briefly, DIvA cells pretreated with the indicated siRNAs were incubated with 4-OHT. Genomic DNA was extracted and digested with *BsrGI* (Figure 1G). The percentage of ssDNA intermediates at the indicated sites was quantified by qPCR. An irrelevant site that spans a *HindIII* restriction site was included as a negative control. Notably, deficiency of NUDT16 led to decreased abundance of ssDNA intermediates (Figure 1H–I). These results are in line with the role of NUDT16 in promoting DSB repair.

### NUDT16 regulates CtIP mediated HR repair.

**Figure 2.**
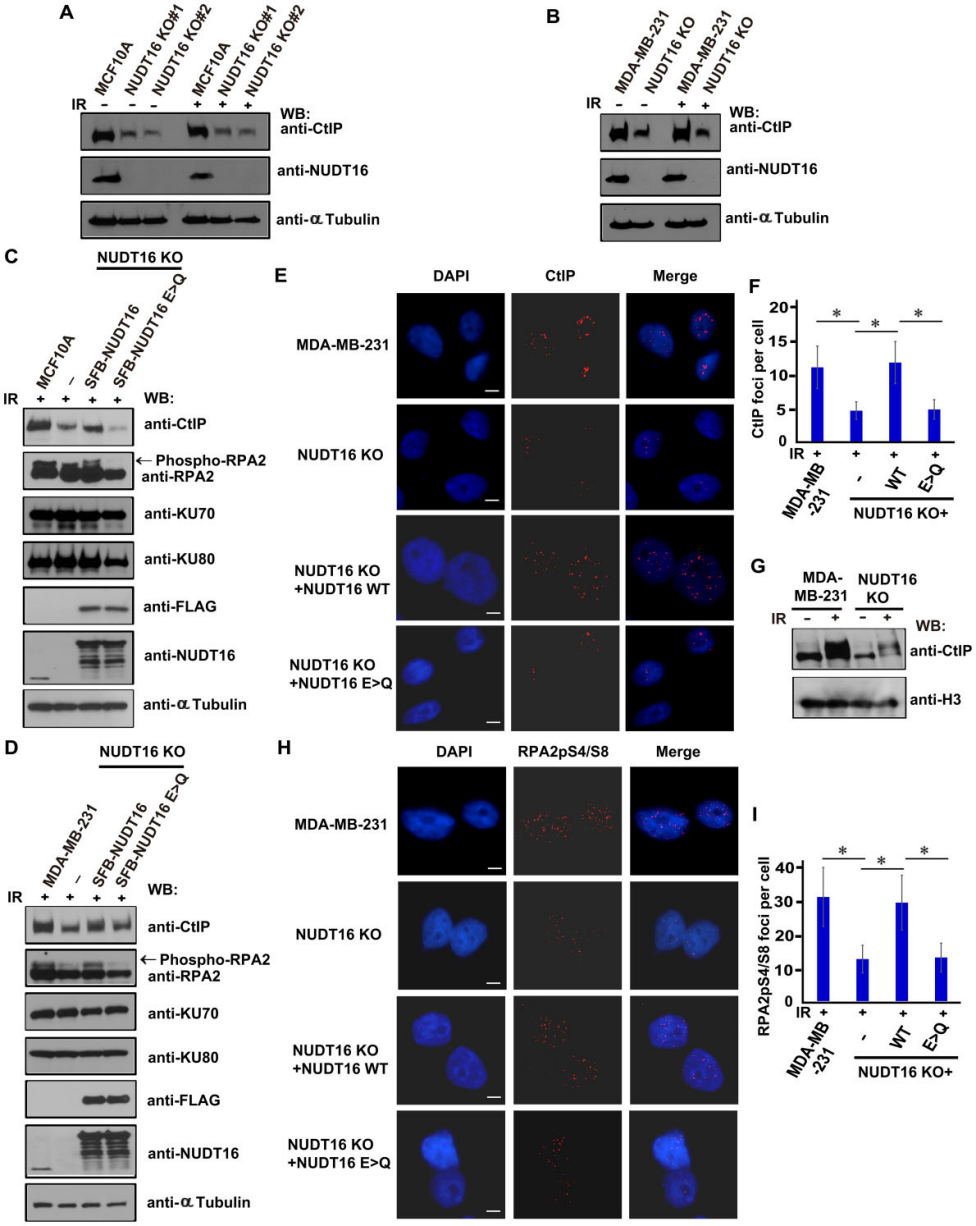
NUDT16 regulates CtIP mediated HR repair. **(A, B)** MCF10A and MCF10A derivative NUDT16 KO cells, or MDA-MB-231 and MDA-MB-231 derivative NUDT16 KO cells were treated with or without IR (20 Gy). Cells were harvested 1 h after IR and immunoblotted with the indicated antibodies. (**C, D**) The catalytic activity of NUDT16 determines protein stability in the HR repair pathway. MCF10A derivative NUDT16 KO cells reconstituted with wild-type (WT) or E > Q mutant of SFB-NUDT16 (**C**) or MDA-MB-231 derivative-derived NUDT16 KO cells reconstituted with WT or E > Q mutant of SFB-NUDT16 (**D**) were harvested at 1 h after IR treatment (20 Gy). The lysates were prepared and immunoblotted with the indicated antibodies. **(E, F)** The catalytic activity of NUDT16 is critical for CtIP localization at DSBs. MDA-MB-231 derivative NUDT16 KO cells reconstituted with WT or E > Q mutant of SFB-NUDT16 were treated with IR (10 Gy). Immunofluorescence was performed using the CtIP antibody at 1 h after IR. (**E**) Representative images of immunofluorescent staining are shown. Scale bar, 10 μm. (**F**) CtIP foci number was quantified (at least 100 cells were counted for each of three independent experiments). Data are represented as the mean ± S.E. (*n* = 3). *P < 0.05. (**G**) Loss of NUDT16 decreases the occupancy of CtIP protein on damaged chromatin. MDA-MB-231 and its derivative NUDT16 KO cells were harvested at 1 h after IR treatment. The chromatin fractions were prepared and immunoblotted with the indicated antibodies. (**H**, **I**) The catalytic activity of NUDT16 regulates RPA2pS4/S8 at DSBs. MDA-MB-231 derivative NUDT16 KO cells reconstituted with WT or E > Q mutant of SFB-NUDT16 were treated with IR (10 Gy). Immunofluorescence was performed using the RPA2pS4/S8 antibody at 1 h after IR. (**H**) Representative images of immunofluorescent staining are shown. Scale bar, 10 μm. (**I**) RPA2pS4/S8 foci number was quantified (at least 100 cells were counted for each of three independent experiments). Data are represented as the mean ± S.E. (*n* = 3). *P < 0.05.

Given that NUDT16 knockdown regulates HR repair efficiency, we hypothesized that NUDT16 may regulate proteins involved in the HR repair pathway. To test this hypothesis, we generated NUDT16 knockout (KO) cells (20), and found that loss of NUDT16 decreased CtIP protein levels in two MCF10A derivative NUDT16 KO cells (Figure 2A) and MDA-MB-231 derivative NUDT16 KO cells (Figure 2B), suggesting that NUDT16 is required for CtIP stability. Moreover, we did not observe significant changes in cell cycle distribution in these cells (Supplemental Figure S1C, D), suggesting that the impact of NUDT16 deficiency on HR repair factor expression is likely not caused by a cell cycle defect. To address whether the hydrolase activity of NUDT16 is required for CtIP protein stability, we generated NUDT16 KO cells with reconstitution of wild-type (WT) NUDT16 or a catalytic inactive E > Q mutant (E76QE79QE80Q) of NUDT16 in both MCF10A cells and MDA-MB-231 cells (20). As shown in Figure 2C, the CtIP protein levels decrease was indeed due to the loss of NUDT16, because only the expression of WT NUDT16 restored the CtIP protein levels in MCF10A derivative NUDT16 KO cells. Moreover, overexpression of NUDT16 E > Q mutant did not rescue the CtIP protein levels in MCF10A derivative NUDT16 KO cells (Figure 2C), demonstrating that a catalytically active NUDT16 is necessary for maintaining CtIP stability. Similar results were observed in MDA-MB-231 derivative NUDT16 KO cells with reconstitution of WT NUDT16 and the E > Q mutant of NUDT16 (Figure 2D, Supplemental Figure S9A). We next assessed whether NUDT16 is critical for CtIP recruitment to DSB sites. The number of CtIP foci was decreased in NUDT16 KO cells after treatment with ionizing radiation (IR), suggesting that loss of NUDT16 impairs CtIP recruitment to DSBs. Moreover, reconstitution of WT NUDT16 restored the CtIP recruitment to DSB sites, whereas the catalytic inactive E > Q mutant of NUDT16 failed to do so (Figure 2E, F). This demonstrates that the hydrolase activity of NUDT16 is important for CtIP accumulation to DSBs. Further corroborating these findings, we found that the loss of NUDT16 reduced CtIP protein levels on chromatin in NUDT16 KO cells, and further decreased the accumulation of CtIP protein on chromatin in response to IR, compared with WT MDA-MB-231 cells (Figure 2G). Taken together, NUDT16 loss impairs CtIP recruitment to DSBs following DNA damage.

DNA end resection is initiated by CtIP and aided by the MRN complex (26–29). The 3′ ssDNA overhangs are quickly bound by RPA to stabilize and protect the ssDNA, and later RPA is replaced by the RAD51 recombinase protein that leads to the search of a homologous template that will achieve accurate HR repair (30,31). Given the effect of NUDT16 on DNA end resection (Figure 1G–I), we questioned whether NUDT16 functions at the level of RPA. Loss of NUDT16 resulted in decreased levels of phosphorylated RPA2 after treatment with IR. The reduction of RPA2 phosphorylation was rescued by overexpression of WT NUDT16, but not with the catalytic inactive E > Q mutant of NUDT16 (Figure 2C, D). RPA2 phosphorylation on the S4 and S8 residues have been used as markers for the generation of single-stranded DNA by DNA-end resection (32,33). Thus, we next asked if the loss of NUDT16 affected RPA2pS4/S8 localization at DSBs. Indeed, RPA2pS4/S8 foci number was decreased in the MDA-MB-231 derivative NUDT16 KO cells after IR (Figure 2H, I). Moreover, the decreased number of RPA2pS4/S8 foci was rescued by overexpression of WT NUDT16, while the E > Q mutant of NUDT16 prevented the localization of RPA2pS4/S8 to DSB sites in NUDT16 KO cells (Figure 2H, I). The heterodimer Ku70/80 is a central player in the c-NHEJ pathway. In contrast to what we observed for CtIP and phosphorylated RPA2, Ku70/Ku80 protein levels were not altered in either cell line after IR (Figure 2C-D). Moreover, NUDT16 inactivation did not alter distal end joining in EJ7-GFP-U2OS reporter cells (34) (Supplemental Figure S1E-S1H). These data suggest that NUDT16 is not involved in c-NHEJ pathway but may participate in the Alt-EJ pathway. Indeed, siRNA mediated knockdown of NUDT16 in EJ2-GFP reporter-containing U2OS cells (22,23) caused a reduction of the GFP-positive cells compared with the control cells (Supplemental Figure S1I-S1L), confirming that NUDT16 participates in the Alt-EJ pathway. Taken together, these results provide evidence that the hydrolase activity of NUDT16 plays a major role in CtIP mediated HR repair.

### NUDT16 removes CtIP ADP-ribosylation

The Nudix enzyme NUDT16 has been shown to have *in vitro* hydrolase activity that removes protein ADP-ribosylation (19,20). Our recent work demonstrated that NUDT16 cleaves the ADP-ribosylation of 53BP1, which inhibits 53BP1 ubiquitination and degradation, and stabilizes 53BP1 protein (20). Therefore, it is reasonable to speculate that the NUDT16 hydrolase activity may be important for regulating ADP-ribosylation of CtIP.

**Figure 3.**
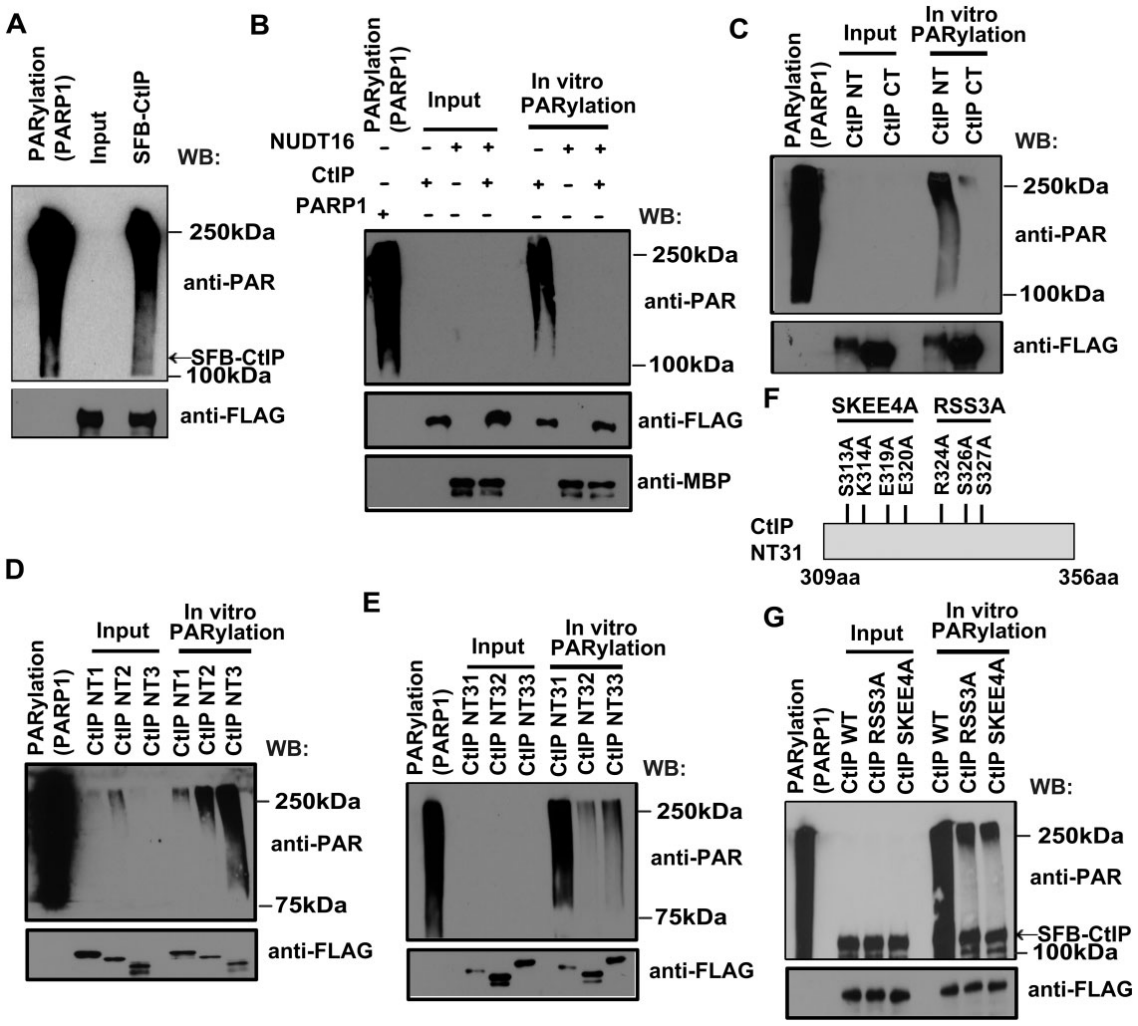
NUDT16 removes CtIP ADP-ribosylation. (**A**) CtIP is ADP-ribosylated. HEK293T cells were transfected with plasmids encoding SFB-tagged CtIP. Cells were lysed with NTEN buffer containing protease inhibitors and phosphatase inhibitors on ice for 30 min. Clear cell lysates were incubated with streptavidin beads at 4°C for 3 h. Then the beads were eluted with 2 mg/ml biotin in NTEN buffer containing protease inhibitors and phosphatase inhibitors at 4°C for 2 h. *In vitro* PARylation assays were performed in a reaction mix consisting of the eluates, 1× reaction buffer, 20 μM NAD+, and 1× active DNA for 30 min at room temperature (RT). PARP1 enzyme serves as a positive control in a reaction mix without eluates. The samples were then immunoblotted with the indicated antibodies. (**B**) NUDT16 removes CtIP ADP-ribosylation. *In vitro* PARylation assays were performed in a reaction mix consisting of SFB-CtIP eluate, 1× reaction buffer, 20μM NAD+ and 1 x active DNA with or without bacterially expressed MBP-NUDT16 protein for 30 min at RT. The samples were immunoblotted with the indicated antibodies. PARP1 enzyme serves as a positive control in a reaction mix without eluates and bacterial expressed MBP-NUDT16 protein. **(C–E)** Mapping the ADP-ribosylation regions of CtIP. *In vitro* PARylation assays were performed as described in (A). Note: NT: 1–450aa; CT: 451–897aa; NT1:1–165aa; NT2:166–308aa; NT3:309–450aa; NT31:309–356aa; NT32:357–404aa; NT33:405–450aa. (**F**) Schematic diagram of mutation sites on CtIP. (**G**) Identification of ADP-ribosylation sites on CtIP. *In vitro* PARylation assays were performed as described in (A). Note: RSS3A: R324AS326AS327A; SKEE4A: S313AK314AE319AE320A.

We first determined whether CtIP is ADP-ribosylated and whether NUDT16 can remove CtIP ADP-ribosylation *in vitro*. We showed that CtIP is ADP-ribosylated by performing an *in vitro* ADP-ribosylation assay (Figure 3A). The PAR signals are only linked to CtIP because CtIP does not interact with PARP1 (Supplemental Figure S2A, B). In addition, we observed that NUDT16 can remove the PAR chains from CtIP (Figure 3B), indicating that CtIP is a substrate of NUDT16 hydrolase activity. To identify the ADP-ribosylated sites in CtIP, we constructed two truncation mutants of CtIP: N-terminus (NT, 1–450 aa) and C-terminus (CT, 451–897aa), and performed the *in vitro* PARylation assay using these mutants. Only the CtIP NT is ADP-ribosylated (Figure 3C). To further narrow down the ADP-ribosylated sites in CtIP, we generated three truncation mutants in the N-terminus of CtIP: NT1 (1–165aa), NT2 (166–308aa) and NT3 (309–450aa), and observed that the NT3 (309–450aa) fragment was ADP-ribosylated (Figure 3D). We performed additional *in vitro* PARylation assays using a series of smaller truncation mutants of CtIP and found that the NT31 (309–356aa) fragment harbors the main residues that are ADP-ribosylated (Figure 3E). Glu (E), Asp (D), Ser (S), Arg (R) and Lys (K) residues have previously been identified as having a high probability of being ADP-ribosylated (35). The NT31 fragment of CtIP was aligned among species, and multiple conserved Glu (E), Lys (K), Ser (S) and Arg (R) residues were identified. Thus, we generated two multi-point mutants (R324AS326AS327A, RSS3A; S313AK314AE319AE320A, SKEE4A) for these conserved residues (Figure 3F) and performed the *in vitro* PARylation assays using these mutants. Both the RSS3A mutant and SKEE4A mutants of CtIP greatly diminished presence of PAR chains (Figure 3G), suggesting that these residues are ADP-ribosylation sites of CtIP.

### DNA damage-induced CtIP ADP-ribosylation leads to CtIP ubiquitination and degradation.

**Figure 4.**
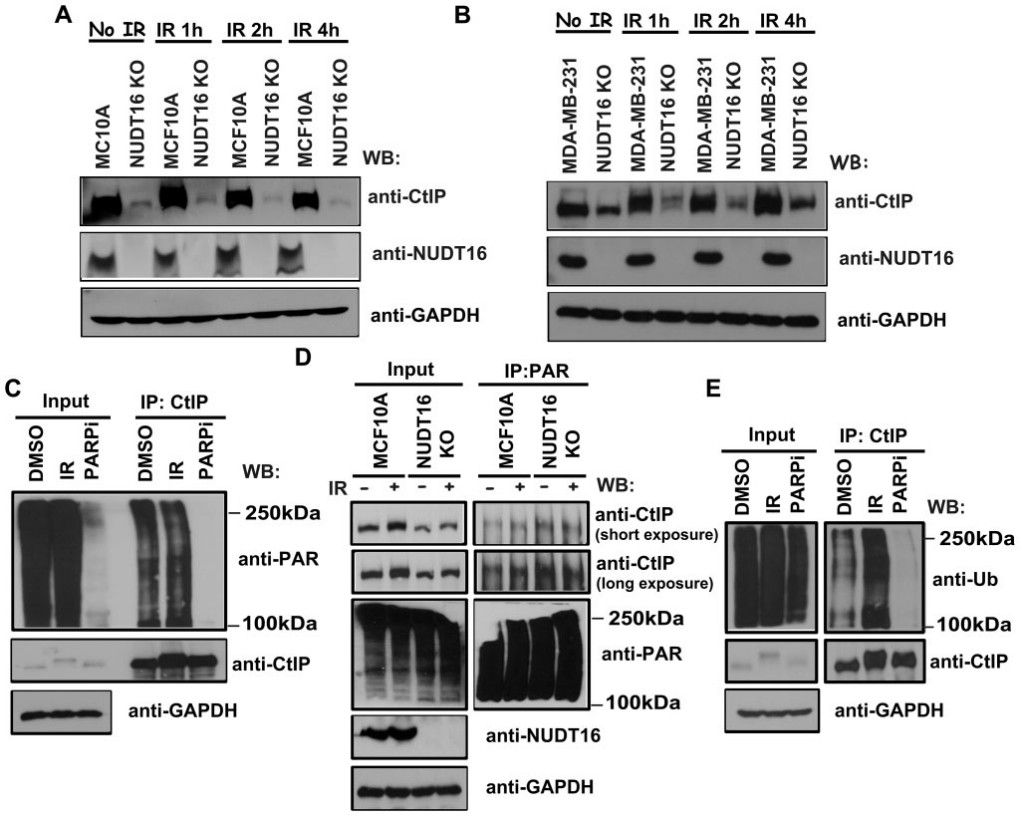
DNA damage-induced CtIP ADP-ribosylation leads to ubiquitin and degradation. **(A, B)** NUDT16 regulates CtIP stability in a time dependent manner in response to DNA damage. MCF10A and MCF10A derivative NUDT16 KO cells (**A**) or MDA-MB-231 and MDA-MB-231 derivative NUDT16 KO cells (**B**) were treated with or without IR (20 Gy). Cells were harvested at the indicated time points and immunoblotted with the indicated antibodies. (**C**) CtIP is ADP-ribosylated *in vivo*. MCF10A cells were mock treated (DMSO) or treated with IR (20 Gy) or PARPi (40 μM olaparib). Cells were harvested 1h after treatment and immunoprecipitated with a CtIP antibody. Immunoprecipitates were blotted using the indicated antibodies. (**D**) Loss of NUDT16 increases ADP-ribosylation of CtIP *in vivo*. MCF10A and MCF10A derivative NUDT16 KO cells were mock treated or treated with IR (20 Gy). Cells were harvested 1h after IR and immunoprecipitated with a PAR antibody. Immunoprecipitates were blotted using the indicated antibodies. (**E**) ADP-ribosylated CtIP mediates its own ubiquitination and degradation. MCF10A cells were mock treated (DMSO) or treated with PARPi (40 μM olaparib) or IR (20 Gy). Cells were harvested at 1 h after treatment and immunoprecipitated with a CtIP antibody. Immunoprecipitates were blotted using the indicated antibodies.

Recent studies have demonstrated that ADP-ribosylation can signal for ubiquitination and promote the degradation of ADP-ribosylated proteins (36–39). Because PARP-dependent protein ADP-ribosylation primes ubiquitination at DNA lesions, we examined the regulation of CtIP protein levels after irradiation-induced DNA damage. After treatment with IR, the CtIP protein levels decreased in MCF10A derivative NUDT16 KO cells in a time-dependent manner, with similar results obtained in MDA-MB-231 derivative NUDT16 KO cells (Figure 4A, B), suggesting that CtIP protein is unstable after DNA damage, possibly due to CtIP ADP-ribosylation-dependent ubiquitination and degradation. To determine whether CtIP is ADP-ribosylated *in vivo*, CtIP was immunoprecipitated with an anti-CtIP antibody, followed by immunoblotting with an antibody to PAR. We observed that CtIP is indeed ADP-ribosylated *in vivo*(Figure 4C, Supplemental Figure S3), and ADP-ribosylated CtIP is slightly increased after IR (Figure 4C). As a control, we treated cells with a Poly (ADP-ribose) Polymerase inhibitor (PARPi, Olaparib) to prevent ADP-ribosylation of CtIP (Figure 4C). Because NUDT16 has hydrolyzing activity that removes protein ADP-ribosylation, we investigated whether CtIP ADP-ribosylation is increased in NUDT16 KO cells. We immunoprecipitated PARylated CtIP protein with anti-PAR antibody, followed by immunoblotting with a CtIP antibody in MCF10A cells and its derivative NUDT16 KO cells. We observed that ADP-ribosylated CtIP is increased in NUDT16 KO cells (Figure 4D, Supplemental Figure S9B), suggesting that NUDT16 has hydrolyzing activity that removes ADP-ribosylation of CtIP *in vivo*. PARPi are used to suppress PARP enzymatic activity to serve as the major barrier to ADP-ribosylation. As shown in Figure 4E, CtIP ubiquitination is drastically increased in response to IR, and we observed a dramatic decrease in CtIP ubiquitination after PARPi treatment. Together, these data suggest that ADP-ribosylated CtIP mediates its own ubiquitination and degradation.

### The E3 ligase RNF146 is required for ADP-ribosylation-dependent ubiquitination and degradation of CtIP

The relationship between ADP-ribosylation and ubiquitination has been well described for the RING-type E3 ubiquitin ligase RNF146, which is responsible for ADP-ribosylation-dependent ubiquitination (40–42). RNF146 contains two conserved domains, a RING domain that is likely an E3 ubiquitin ligase, and a WWE domain that is a PAR-binding domain (43).

**Figure 5.**
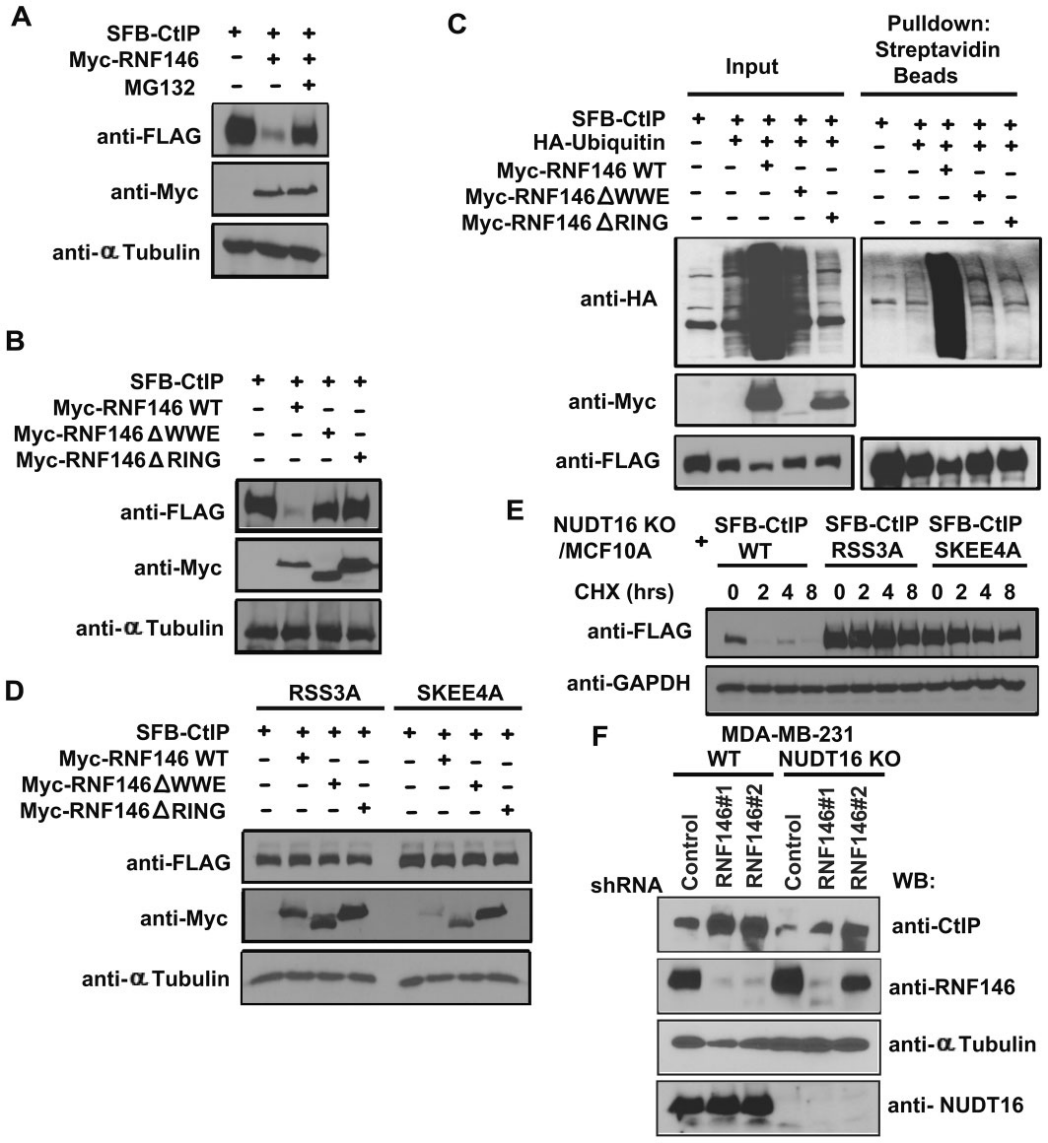
The E3 ligase RNF146 is required for ADP-ribosylation-dependent ubiquitination and degradation of CtIP. (**A**) RNF146 leads to reduced CtIP protein. HEK293T cells were transfected with constructs encoding SFB-CtIP together with vector alone or construct encoding Myc-tagged wild-type (WT) RNF146. 24 h after transfection, cells were treated with the proteasome inhibitor MG132 (20 μM) for 4 h. Lysates were prepared and examined by Western blotting using the indicated antibodies. (**B**) The WWE domain and RING domain of RNF146 are required for CtIP ubiquitination and degradation. HEK293T cells were transfected with constructs encoding SFB-CtIP together with vector alone or constructs encoding Myc-tagged WT RNF146, the RNF146-ΔWWE mutant, or the RNF146-ΔRING mutant. Lysates were prepared and examined by Western blotting using the indicated antibodies. (**C**) RNF146 promotes ubiquitination of CtIP. HEK293T cells were transfected with constructs encoding HA-tagged ubiquitin, SFB-tagged CtIP, and Myc-tagged WT RNF146, the ΔWWE mutant of RNF146, or the ΔRING mutant of RNF146. 24 h after transfection, cells were treated with the proteasome inhibitor MG132 (20μM) for 4 h. Lysates were prepared and analyzed by immunoprecipitation/Western blotting using the indicated antibodies. (**D**) RNF146 does not facilitate the degradation of the RSS3A or SKEE4A mutant of SFB-CtIP. HEK293T cells were transfected with constructs encoding the RSS3A or SKEE4A mutant of CtIP together with vector alone, or with constructs encoding Myc-tagged WT RNF146, the RNF146-ΔWWE mutant, or the RNF146-ΔRING mutant. Lysates were prepared and examined by Western blotting using the indicated antibodies. (**E**) Loss of CtIP ADP-ribosylation enhances CtIP protein stability. MCF10A derived NUDT16 KO cells were reconstituted with WT or RSS3A or SKEE4A mutants of SFB-CtIP. After treatment with cycloheximide (CHX, 10μg/ml), cells were harvested at the indicated timepoints and immediately lysed with 2× Laemmli buffer. Lysates were immunoblotted with the indicated antibodies. (**F**) RNF146 depletion led to CtIP stability. RNF146 knockdown was performed in MDA-MB-231 and its derivative NUDT16 KO cells. Lysates were prepared and examined by western blotting.

We tested whether RNF146 also acts as an E3 ubiquitin ligase for ADP-ribosylated CtIP. Expression of WT RNF146 led to reduced CtIP protein levels, which was completely reversed by the proteasome inhibitor MG132 (Figure 5A). Next, we examined whether the WWE domain or RING domain of RNF146 affects the CtIP ADP-ribosylation. RNF146ΔWWE or RNF146ΔRING mutant had no effect on CtIP protein levels (Figure 5B), suggesting that CtIP needs to be ADP-ribosylated before RNF146 can recognize it for ubiquitination and degradation. Indeed, only WT RNF146 promoted the ubiquitination of CtIP (Figure 5C), indicating that both the PAR recognition domain (WWE) and the E3 ligase activity domain (RING) of RNF146 are required for RNF146-mediated CtIP ubiquitination and degradation. However, we did not observe the RNF146-CtIP interaction by endogenous IP and Co-IP (Supplemental Figure S2C, D), indicating that RNF146 does not bind to CtIP, but can recognize and target PARylated CtIP. To determine the effect of the ADP-ribosylated residues of CtIP on RNF146 mediated CtIP ubiquitination and degradation, we co-transfected the RSS3A or SKEE4A mutants of CtIP with RNF146 WT, RNF146ΔWWE, or RNF146ΔRING mutant. RNF146 failed to facilitate the degradation of the RSS3A or SKEE4A mutant of CtIP (Figure 5D), again supporting the idea that RNF146-mediated CtIP ubiquitination and degradation is dependent on CtIP being ADP-ribosylated.

We also performed cycloheximide treatment experiments and showed that the half-life of WT CtIP was significantly shorter than that of the RSS3A or SKEE4A mutant of CtIP in MCF10A derived NUDT16 KO cells, indicating that loss of CtIP ADP-ribosylation enhances CtIP protein stability (Figure 5E). Additionally, the protein levels for the RSS3A or SKEE4A mutants of CtIP was stable in NUDT16 KO cells, suggesting that mechanistically, CtIP stability depends on ADP-ribosylation-dependent ubiquitination and degradation. To address whether RNF146 regulates the degradation of CtIP and if NUDT16 is involved in this process, we depleted endogenous RNF146 in both MDA-MB-231 and its derivative NUDT16 KO cells using shRNA knockdown. We observed that depletion of endogenous RNF146 resulted in stabilization of CtIP in both MDA-MB-231 and its derivative NUDT16 KO cells (Figure 5F, Supplemental Figure S9C), demonstrating that NUDT16 loss promotes ADP-ribosylation of CtIP, which in turn leads to its ubiquitination and degradation by the E3 ligase RNF146.

### ADP-ribosylation of CtIP is required for its binding to NUDT16 and for its participation in the DNA damage response

**Figure 6.**
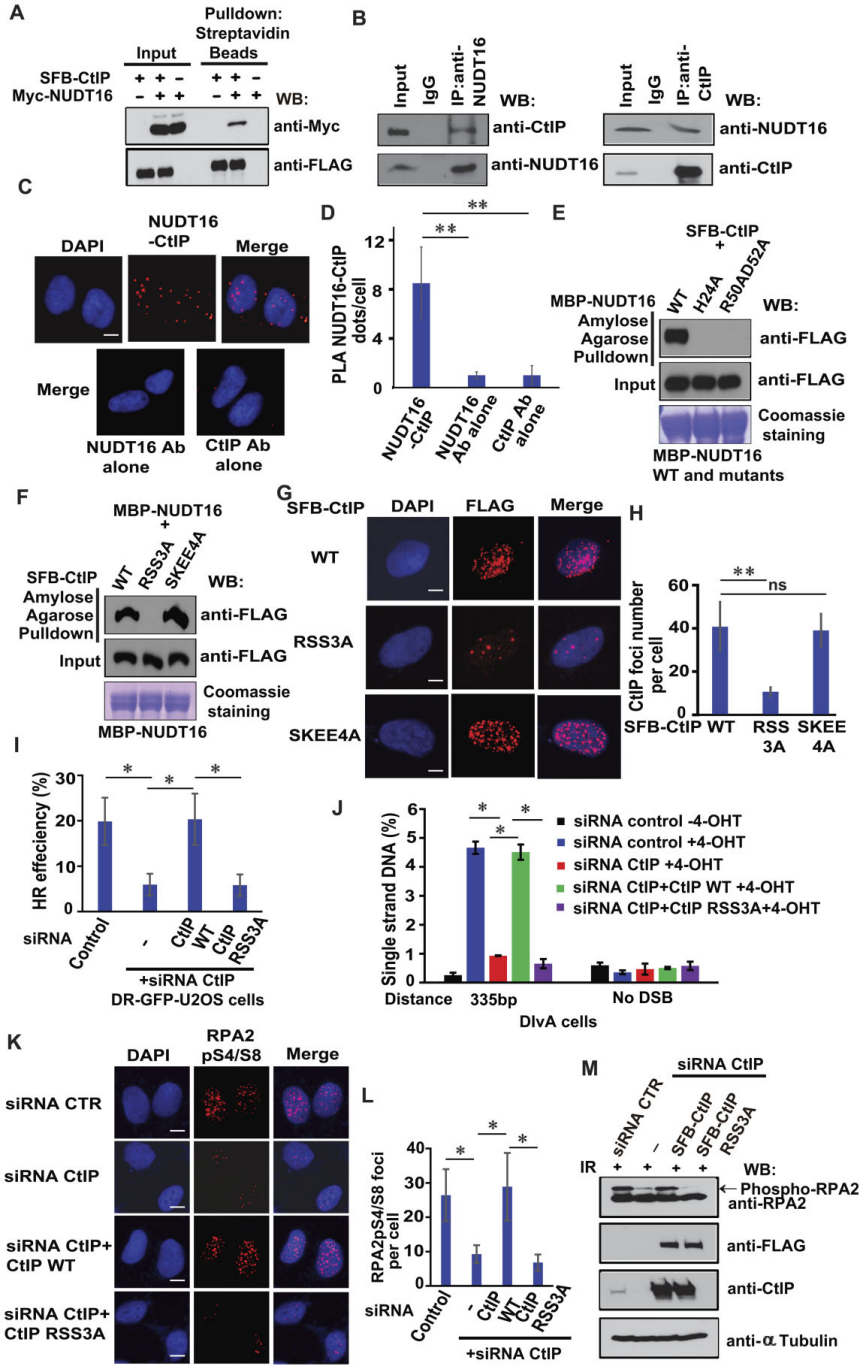
CtIP is a novel NUDT16-interacting protein. (**A**) NUDT16 interacts with CtIP by Co-IP experiment. HEK293T cells were transfected with plasmids encoding SFB-tagged CtIP or Myc-tagged NUDT16. Co-IP reactions were performed with streptavidin beads and subjected to western blotting with the indicated antibodies. (**B**) Endogenous NUDT16 interacts with CtIP. HEK293T cell lysates were prepared and immunoprecipitated with NUDT16 antibody (left panel) or CtIP antibody (right panel) followed by immunoblotting with the CtIP antibody or NUDT16 antibody, respectively. (**C**) PLA detection of the NUDT16-CtIP interaction. U2OS cells were subjected to PLA using the NUDT16 and CtIP antibodies, shown as distinct fluorescent dots. Negative controls used one of these two antibodies. Scale bar, 10 μm. (**D**) Quantification of the results in (**C**). PLA dots per cell were quantified in 50 cells for each experiment. Data are represented as the mean ± S.E. (*n* = 3). **P < 0.01. (**E**) His24, Arg50 and Asp52 residues of NUDT16 are required for the NUDT16-CtIP interaction. Beads coated with bacterially expressed and purified MBP-NUDT16 WT, H24A or R50AD52A fusion proteins were incubated, respectively, with cell lysates containing exogenously expressed SFB-CtIP. Immunoblotting experiments were carried out using the indicated antibodies. (**F**) The RSS3A mutant of CtIP abolishes the NUDT16-CtIP interaction. Beads coated with bacterially expressed and purified MBP-NUDT16 fusion protein were incubated with cell lysates containing exogenously expressed SFB-CtIP WT, RSS3A, or SKEE4A mutant. Immunoblotting experiments were carried out using the indicated antibodies. (**G**) ADP-ribosylation of CtIP is required for the CtIP localization at DSBs. U2OS cells were transfected with plasmids encoding WT, RSS3A or SKEE4A mutant of SFB-CtIP, respectively, then followed by IR (10 Gy). One hour later, the cells were fixed and immunostained with anti-FLAG antibody. (**H**) Quantification of the results in (G). The CtIP foci number per nucleus were quantified in 50 cells for each experiment. Data are represented as the mean ± S.E. (*n* = 3). **P < 0.01; ns: not significant. (**I**) Defect in CtIP PARylation impairs HR repair. DR-GFP-U2OS cells were reconstituted with siRNA-resistant WT or RSS3A mutant of SFB-tagged CtIP. After CtIP depletion using siRNA for 24 h, these cells were transfected with I-SceI plasmid and pcDNA3.1-mCherry at 9:1 ratio for another 48 h. The GFP^+^ and mCherry^+^ cell population was quantitated, and the HR rate represented as GFP^+^/mCherry^+^. Data are represented as the mean ± S.E. (*n* = 3). *P < 0.05. (**J**) CtIP ADP-ribosylation affects DNA end resection activity. DIvA cells were reconstituted with siRNA-resistant WT or RSS3A mutant of SFB-tagged CtIP. These cells pretreated with CtIP siRNA were incubated with 4-OHT for 4 h. Genomic DNA was extracted and digested with *BsrGI* or *HindIII*. The percentage of ssDNA intermediates at indicated sites was measured by qPCR. Data represents mean ± S.E. (*n* = 3) experiments. *P < 0.05. (**K–L**) CtIP ADP-ribosylation regulates RPA2pS4/S8 at DSBs. U2OS cells were reconstituted with siRNA-resistant WT or RSS3A mutant of SFB-tagged CtIP. After CtIP depletion using siRNA for 48 h, these cells were treated with IR (10 Gy). Immunofluorescence was performed using the RPA2pS4/S8 antibody at 1 h after IR. (**K**) Representative images of immunofluorescent staining are shown. Scale bar, 10 μm. (**L**) RPA2pS4/S8 foci number was quantified (at least 50 cells were counted for each of three independent experiments). Data are represented as the mean ± S.E. (*n* = 3). *P < 0.05. (**M**) CtIP ADP-ribosylation affects RPA2 protein level following DNA damage. U2OS cells were reconstituted with siRNA-resistant WT or RSS3A mutant of SFB-tagged CtIP. After CtIP depletion using siRNA for 48 h, these cells were treated with IR (20 Gy). Cells were harvested 1 h after IR and immunoblotted with the indicated antibodies.

We sought to determine whether NUDT16 interacts with CtIP. We performed co-immunoprecipitation experiments, and observed that NUDT16 interacts with CtIP (Figure 6A). Moreover, we confirmed that the NUDT16–CtIP interaction occurs between endogenous proteins by immunoprecipitation using both a NUDT16 antibody and a CtIP antibody (Figure 6B). This suggests that these two proteins indeed associate with each other *in vivo*. Next, we performed the *in situ* proximity ligation assay (PLA), which allows direct visualization, as well as quantification, of proteins that are in close vicinity. PLA detection of the NUDT16-CtIP interaction was visualized as distinct fluorescent dots in U2OS cells (Figure 6C, D). This interaction was specific, since only very few PLA dots were observed when the NUDT16 antibody or CtIP antibody was used alone, or when NUDT16 or CtIP was depleted (Figure 6C, D, Supplemental Figure S4A–F). Collectively, these results provide support for a physical interaction between NUDT16 and CtIP.

To determine the regions on NUDT16 required for its interaction with CtIP, we subjected bacterially expressed and purified WT NUDT16 and a series of NUDT16 internal-deletion mutants to perform pulldown experiments with SFB-tagged CtIP. We found that deletion of the N-terminus of NUDT16 abolishes the NUDT16-CtIP interaction, while deletion of the C-terminus of NUDT16 only decreased the NUDT16-CtIP interaction (Supplemental Figure S4G), indicating that the N-terminus of NUDT16 is the major region involved in the NUDT16-CtIP interaction, while the C-terminus of NUDT16 only contributes to this interaction. Previous reports demonstrate that His24, Arg50 and Asp52 within the N-terminus of NUDT16 likely participate in substrate recognition (44). Thus, we mutated these three residues in the N-terminus of NUDT16 (H24A and R50AD52A). A pulldown experiment showed that these mutants disrupted the NUDT16-CtIP interaction (Figure 6E). Next, we sought to define the NUDT16 binding sites on CtIP. Because we identified several PARylation sites on CtIP (Figure 3G), we performed a pulldown experiment using bacterially expressed and purified NUDT16 protein incubated with overexpressed SFB-CtIP or its mutants. We observed that the RSS3A mutant of CtIP disrupted the NUDT16-CtIP interaction, whereas the SKEE4A mutant had no effect on the NUDT16-CtIP interaction (Figure 6F). These results indicate that the ADP-ribosylation of CtIP is responsible for the NUDT16-CtIP interaction.

To further confirm the specificity of the NUDT16-CtIP interaction, the PLA assay was performed in U2OS cells. In agreement with the Co-IP, the NUDT16-CtIP interaction was also detected *in situ* by PLA. A significant lower number of PLA dots per cell was observed with the H24A or R50AD52A mutant of NDUT16, compared to cells transfected with WT NUDT16 (Supplemental Figure S4H-S4I). Furthermore, cells transfected with a CtIP RSS3A mutant displayed much fewer dots per cell (Supplemental Figure S4J-S4K), showing the specific involvement of the ADP-ribosylation of CtIP in the interaction of NUDT16 and CtIP. Taken together, these data suggest that NUDT16 interacts with CtIP, and that the NUDT16-CtIP interaction is mediated by the His24, Arg50 or Asp52 of NUDT16 and the ADP-ribosylation sites (Arg324, Ser326 or Ser327) of CtIP.

In mammalian cells, CtIP is recruited to DNA damage sites induced by IR or laser irradiation (26,45–47). To determine whether ADP-ribosylation of CtIP affects its localization at DSBs, U2OS cells were transfected with WT, RSS3A, or SKEE4A mutants of SFB-CtIP. IR-induced CtIP focus formation was observed in the cells expressing WT and SKEE4A mutant of CtIP, but the number of foci was significantly decreased in cells expressing the RSS3A mutant of CtIP (Figure 6G, H), demonstrating that PARylation deficient RSS3A mutant impairs the CtIP focus formation after irradiation. Because the Ser327 is required for BRCA1-CtIP interaction and CtIP recruitment to DSBs (46,48). In this regard, it is reasonable that the number of foci was significantly decreased in cells expressing the RSS3A mutant of CtIP because the Ser327 is within the residues Arg324, Ser326 and Ser327 (RSS) of CtIP (Figure 6G, H). To further dissect the role of these three residues in CtIP PARylation and its recruitment to DSBs, we generated R324AS326A (RS2A) and Ser327A mutants of CtIP. Both the RS2A and S327A mutants of CtIP greatly diminished PAR chains (Supplemental Figure S5A). In addition, the number of foci was reduced in cells expressing the RS2A mutant and the S327A mutant of CtIP (Supplemental Figure S5B, C), suggesting that all three residues contribute to the CtIP PARylation and its recruitment to DSBs. Next, to determine whether CtIP PARylation affects cellular sensitivity to IR, we performed clonogenic survival assays. Cell survival following IR treatment of U2OS cells with CtIP knockdown demonstrated that the loss of CtIP resultd in increased sensitivity to IR. Moreover, reconstitution of WT CtIP decreased cell sensitivity to IR; however, reconstitution with the RSS3A mutant of CtIP failed to do so (Supplemental Figure S5D), indicating that this PARylation deficient RSS3A mutant of CtIP leads to IR sensitivity. To investigate whether the RSS3A mutant of CtIP that abrogates ADP-ribosylation exhibits HR repair and DNA end-resection defects, DR-GFP-U2OS or DIvA cells were reconstituted with siRNA-resistant WT or a RSS3A mutant of SFB-tagged CtIP, so that we can express exogenous CtIP when the endogenous CtIP is depleted by siRNA. The HR repair or DNA end resection activity is reduced when CtIP is depleted. Reconstitution of wild-type CtIP restored HR repair or DNA end resection activity, while the RSS3A mutant of CtIP failed to do so, indicating that PARylation deficient RSS3A mutant of CtIP impairs HR repair and DNA end resection (Figure 6I, J, Supplemental Figure S5E–G). Furthermore, we observed that RPA2pS4/S8 foci number was decreased in the CtIP depleted U2OS cells after IR (Figure 6K, L), and the decreased number of RPA2pS4/S8 foci was rescued by overexpression of WT CtIP, while the RSS3A mutant of CtIP prevented the localization of RPA2pS4/S8 to sites of DNA damage in CtIP depleted cells (Figure 6K, L). Further corroborating these findings, we found that the reduction of RPA2 phosphorylation by CtIP depletion was restored by overexpression of WT CtIP, but not with the RSS3A mutant of CtIP (Figure 6M). These data indicate that the Arg324, Ser326 or Ser327 residues of CtIP, which are required for its ADP-ribosylation, are important for the function of CtIP in response to DNA damage and its DNA repair.

### NUDT16 ADP-ribosylation is important for its function in HR repair

**Figure 7.**
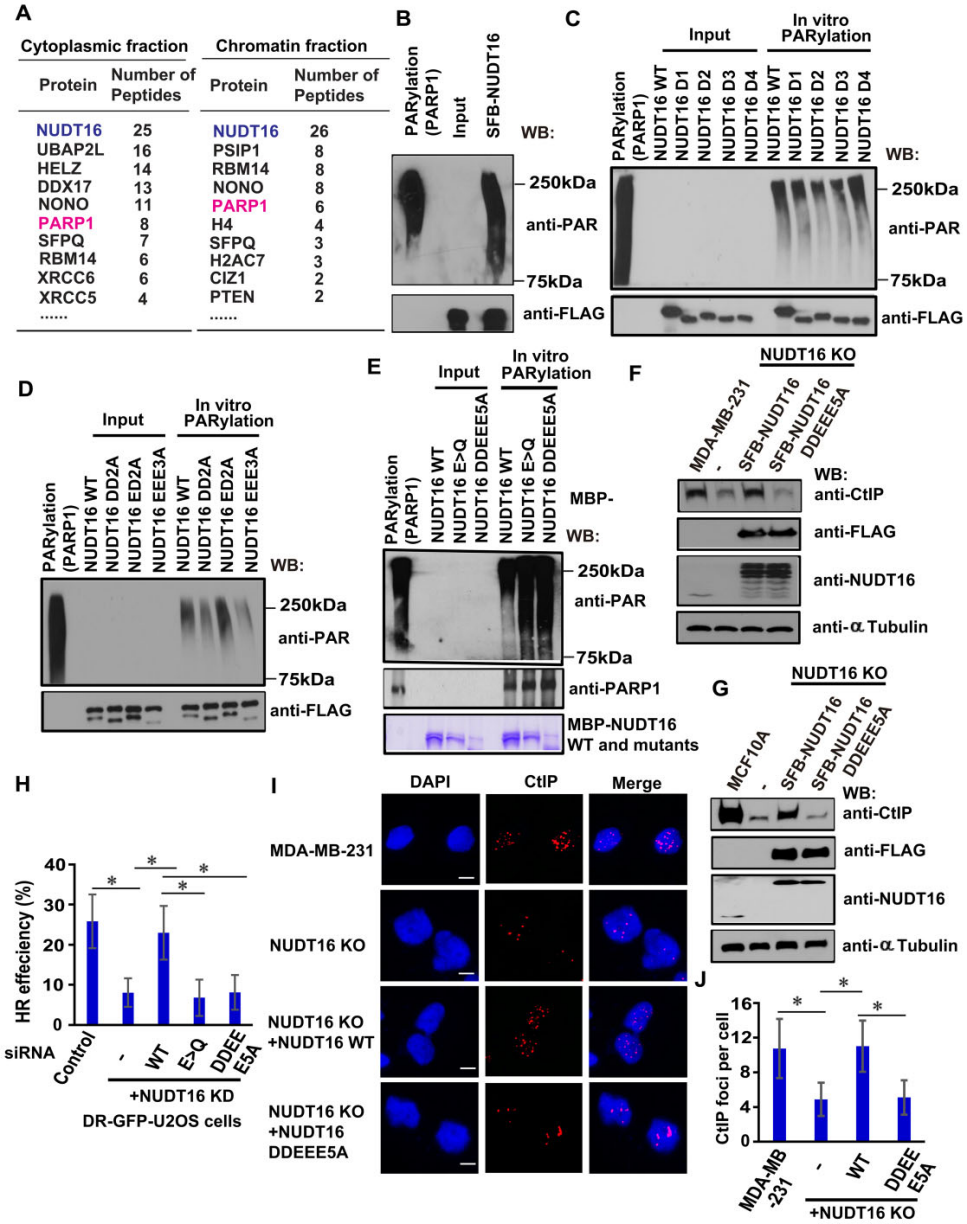
NUDT16 ADP-ribosylation is important for its function in HR repair. (**A**) PARP1 is identified as a NUDT16-associated protein in the cytoplasmic and chromatin fractions by mass spectrometric analysis. (**B**) NUDT16 is ADP-ribosylated. The *in vitro* PARylation assays were performed as described in Figure 3A. (**C**) Map of the ADP-ribosylation regions on CtIP. The *in vitro* PARylation assays were performed as described in Figure 3A. Note: The schematic diagram of NUDT16 WT/D1/D2/D3/D4 is shown in Supplemental Figure S7J. (**D**) Identification of ADP-ribosylation sites on NUDT16. *In vitro* PARylation assays were performed as described in Figure 3A. Note: DD2A: D63AD66A; ED2A: E91AD94A; EEE3A: E118AE119AE124A. (**E**) The hydrolase activity of NUDT16 is attenuated in the DDEEE5A mutant. *In vitro* PARylation assays were performed in a reaction mix consisting of bacterially expressed proteins of NUDT16 (WT, DDEEE5A mutant, or E > Q mutant), PARP1 enzyme, 1× reaction buffer, 20 μM NAD+, and 1x active DNA for 30 min at room temperature. PARP1 enzyme serves as a positive control in a reaction mix without NUDT16 proteins. The samples were immunoblotted with the indicated antibodies. Note: E > Q: E76QE79QE80Q; DDEEE5A: D63AD66AE118AE119AE124A. **(F, G)** The ADP-ribosylation of NUDT16 regulates CtIP protein stability. MDA-MB-231 (**F**) or MCF10A (**G**) derivative NUDT16 KO cells reconstituted with WT or DDEEE5A mutant of SFB-NUDT16 were harvested at 1 h after IR treatment (20 Gy). The lysates were prepared and immunoblotted with the indicated antibodies. (**H**) NUDT16 PARylation participates in HR repair. DR-GFP-U2OS cells were reconstituted with the siRNA-resistant wild-type, E > Q mutant, or DDEEE5A mutant of SFB-tagged NUDT16. After NUDT16 depletion using siRNA for 24 h, these cells were transfected with I-SceI plasmid and pcDNA3.1-mCherry at 9:1 ratio for another 48 h. The GFP^+^ and mCherry^+^ cell population was quantitated. Data are represented as the mean ± S.E. (*n* = 3). *P < 0.05. **(I, J)** The ADP-ribosylation of NUDT16 is critical for CtIP localization at DSBs. MDA-MB-231 derivative NUDT16 KO cells reconstituted with WT or DDEEE5A mutant of SFB-NUDT16 were treated with IR (10 Gy). Immunofluorescence was performed using the CtIP antibody at 1 h after IR. (**I**) Representative images are shown by immunofluorescent staining. Scale bar, 10 μm. (**J**) CtIP foci number was quantified (at least 50 cells were counted from three independent experiments). Data are represented as the mean ± S.E. (*n* = 3). *P < 0.05.

To further understand how NUDT16 function in DNA damage response is regulated, we performed tandem affinity purification (TAP) using cytoplasmic and chromatin fractions derived from HEK293T cells stably expressing the SFB-tagged NUDT16. Mass spectrometry analysis revealed that PARP1 is a NUDT16-associated protein in both cytoplasmic and chromatin fractions (Figure 7A). Because PARP-1 catalyzes ADP-ribosylation of the acceptor proteins, we first set out to determine whether NUDT16 is ADP-ribosylated by PARP1 *in vitro*. Indeed, NUDT16 is ADP-ribosylated, as shown by an *in vitro* ADP-ribosylation assay (Figure 7B). Furthermore, NUDT16 is ADP-ribosylated by PARP1 (Supplemental Figure S6A). PARPi are used to inhibit PARP enzymatic activity, which serves as a major barrier to ADP-ribosylation. After treatment with PARPi, NUDT16 PARylation is greatly diminished (Supplemental Figure S6A). To identify the ADP-ribosylated sites in NUDT16, we performed the ADP-ribosylation assay using the deletion mutants of NUDT16 (Supplemental Figure S7J) and found the strongest ADP-ribosylation on the D4 mutant of NUDT16 (Figure 7C). We then constructed two truncation mutants of NUDT16: N-terminus (NT1, 1–62 aa) and C-terminus (NT2, 63–130aa), and revealed that the NUDT16 NT2 is major region for ADP-ribosylation (Supplemental Figure S6B). The NT2 fragment of NUDT16 was aligned among species, and multiple conserved Glu (E) and Asp (D) residues were identified. Thus, we generated three multi-point mutants (D63AD66A, DD2A; E91AD94A, ED2A; E118AE119AE124A, EEE3A) for these conserved residues and again performed the *in vitro* PARylation assay. Both the DD2A mutant and the EEE3A mutant of NUDT16 substantially reduced PAR chains (Figure 7D), strongly suggesting that these residues are potential ADP-ribosylation sites of NUDT16. Interestingly, the catalytically inactive mutant (E > Q or E > A) of NUDT16 retained a strong PARylation signal (Supplemental Figure S6C), suggesting that ADP-ribosylated NUDT16 is stable due to loss of hydrolase activity (E > Q or E > A) in NUDT16. Thus, when we generated the DD2A or the EEE3A mutant in the catalytically inactive E > A mutant of NUDT16, the ADP-ribosylation signal was dramatically reduced in the DD2A, EEE3A or DD2A/EEE3A (DDEEE5A) mutants of NUDT16 (Supplemental Figure S6D), further confirming DDEEE residues of NUDT16 are ADP-ribosylation sites of NUDT16.

Next, we attempted to examine whether the DDEEE5A mutant of NUDT16 affects the hydrolase activity that removes PAR chains. We performed the *in vitro* PARylation assay and observed that the DDEEE5A and E > Q mutants of NUDT16 failed to remove the PAR chains from PARP1 (Figure 7E), indicating that the DDEEE are catalytically active residues required for NUDT16 hydrolase activity. To examine whether the ADP-ribosylation of NUDT16 regulates CtIP protein level, we generated NUDT16 knockout (KO) cells (20), as well as NUDT16 KO cells with reconstituted WT NUDT16 and the PARylation deficient mutant (DDEEE5A) of NUDT16 in MDA-MB-231 and MCF10A cells. As shown in Figure 7F, G, the expression of WT NUDT16 restored the CtIP protein levels in MCF10A or MDA-MB-231 derivative NUDT16 KO cells, whereas overexpression of the NUDT16 DDEEE5A mutant did not rescue the CtIP protein levels in MDA-MB-231 or MCF10A derivative NUDT16 KO cells. Notably, the expression of WT NUDT16 restored the HR repair capacity in NUDT16 depleted cells, whereas overexpression of the NUDT16 DDEEE5A mutant did not rescue the HR repair capacity in NUDT16 depleted cells (Figure 7H and Supplemental Figure S6E, F), indicating that NUDT16 PARylation affects its HR repair function. We next assessed whether ADP-ribosylation of NUDT16 is critical for CtIP recruitment to DSBs. CtIP foci number was decreased in NUDT16 KO cells after treatment with IR, and reconstitution of WT NUDT16 restored CtIP recruitment to DSB sites. In contrast, the PARylation deficient mutant (DDEEE5A) of NUDT16 failed to do so (Figure 7I-J), demonstrating that ADP-ribosylation of NUDT16 is important for CtIP accumulation at DSBs.

### The PARP1–NUDT16 interaction is regulated by IR-induced DNA damage.

**Figure 8.**
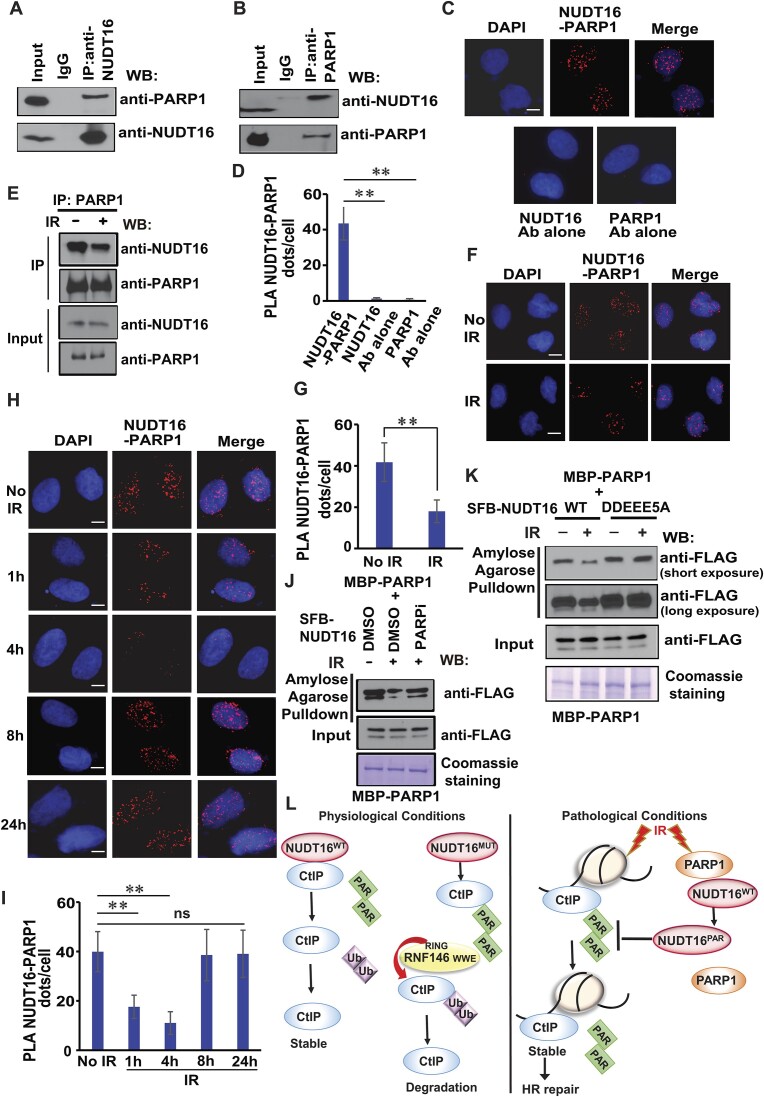
The NUDT16-PARP1 interaction is regulated by IR-induced DNA damage. **(A, B)** Endogenous NUDT16 interacts with PARP1. MCF10A cell lysates were prepared and immunoprecipitated with NUDT16 antibody (**A**) or PARP1 antibody (**B**) followed by immunoblotting with the PARP1 or NUDT16 antibody, respectively. **(C, D)** PLA detection of the NUDT16-PARP1 interaction. U2OS cells were subjected to the PLA assay using the NUDT16 and PARP1 antibodies, shown as distinct fluorescent dots. Negative controls used one of these two antibodies. Scale bar, 10 μm. (**D**) Quantification of the results in (**C**). PLA dots per cell were quantified in 100 cells for each experiment. Data are represented as the mean ± S.E. (*n* = 3). **P< 0.01. (**E**) The NUDT16-PARP1 interaction was reduced after IR. MCF10A cell lysates were either left untreated or irradiated with 20 Gy for 1 h. Cell lysates were prepared and immunoprecipitated with PARP1 antibody followed by immunoblotting with the indicated antibodies. **(F, G)** The NUDT16-PARP1 interaction is regulated by IR-induced DNA damage. U2OS cells were either left untreated or irradiated with 10 Gy. One hour later, a PLA assay was performed using the NUDT16 and PARP1 antibodies, shown as distinct fluorescent dots. Scale bar, 10 μm. (**G**) Quantification of the results in (**F**). PLA dots per cell were quantified in 100 cells for each experiment. Data are represented as the mean ± S.E. (*n* = 3). **P< 0.01. **(H, I)** The PARP1-NUDT16 interaction is regulated by IR-induced DNA damage in a time-dependent manner. U2OS cells were either left untreated or irradiated with 10 Gy. (**H**) A PLA assay was performed at the indicated time points using the NUDT16 and PARP1 antibodies, shown as distinct fluorescent dots. Scale bar, 10 μm. (**I**) Quantification of the results in (**H**). PLA dots per cell were quantified in 100 cells for each experiment. Data are represented as the mean ± S.E. (*n* = 3). **P< 0.01; ns: not significant. (**J**) The NUDT16–PARP1 interaction is regulated by DNA damage and PARPi by MBP pulldown assay. HEK293T cells with expressed SFB-NUDT16 were either left untreated, irradiated with 20 Gy, or treated with PARPi (20μM olaparib) for 3 h prior to irradiation with 20 Gy. One hour later, beads coated with bacterially expressed MBP-PARP1 fusion protein were incubated with cell lysates containing exogenously expressed SFB-NUDT16. Immunoblotting experiments were carried out using the indicated antibodies. (**K**) The NUDT16–PARP1 interaction is regulated by ADP-ribosylation of NUDT16. HEK293T cells expressing WT, DDEEE5A mutant of SFB-NUDT16 were either left untreated or irradiated with 20 Gy. One hour later, beads coated with bacterially expressed MBP-PARP1 fusion protein were incubated, respectively, with cell lysates containing exogenously expressed WT, DDEEE5A mutant of SFB-NUDT16. Immunoblotting experiments were carried out using the indicated antibodies. (**L**) A working model representing the regulation of CtIP by NUDT16 and its ADP-ribosylation. Please see details in the *Discussion*. Note: NUDT16^WT^: wild-type NUDT16; NUDT16^MUT^: NUDT16 E > Q mutant, NUDT16 DDEEE5A mutant, or NUDT16 KO; NUDT16^PAR^: NUDT16 PARylation.

As shown in Figure 7A, PARP1 is likely a NUDT16-associated protein. We confirmed that PARP1 interacts with NUDT16 through immunoprecipitation experiments using a NUDT16 antibody and a PARP1 antibody (Figure 8A, B), respectively, suggesting that these two proteins do indeed interact with each other *in vivo*. Of note, the NUDT16–PARP1 interaction was not affected when the extracts were treated with the Benzonase nuclease, suggesting that the interaction is not dependent upon the presence of DNA (Supplemental Figure S7A, B). Moreover, the PARP1–NUDT16 interaction was also detected *in situ* by the PLA assay (Figure 8C, D, Supplemental Figure S7C–E). It has been shown that PARP1 facilitates Alt-NHEJ repair (49). We next investigated how the PARP1–NUDT16 interaction may be regulated after DNA damage. In this regard, an endogenous immunoprecipitation was performed in MCF10A cells either left untreated or treated with IR. We found that the interaction of PARP1 with NUDT16 was reduced at 1 h after IR (Figure 8E). In agreement with the IP results, we also observed that the PARP1-NUDT16 interaction was reduced under IR conditions as determined by the PLA assay (Figure 8F, G). Moreover, the PARP1-NUDT16 interaction was decreased at the early timepoint (1 and 4 h) in a PLA assay (Figure 8H, I). However, we observed that the PARP1-NUDT16 interaction at 8 and 24 h after IR was similar to that of the untreated cells. Taken together, these data suggest that NUDT16 interacts with PARP1, and that the PARP1-NUDT16 interaction is regulated by IR-induced DNA damage in a time-dependent manner.

Because NUDT16 is ADP-ribosylated by PARP1 (Figure 7B and Supplemental Figure S6A), we investigated whether the NUDT16-PARP1 interaction is regulated by NUDT16 PARylation. PARP inhibitor (PARPi) is used to inhibit PARP enzymatic activity and serves as the major barrier to ADP-ribosylation. In this regard, an MBP pull-down assay was performed in HEK293T cells expressing SFB-NUDT16 either left untreated, treated with IR, or treated with PARPi prior to IR. We found that the interaction of MBP-PARP1 with SFB-NUDT16 was reduced by IR, and this IR-induced decreased interaction is prevented if cells were treated with the PARPi (Figure 8J), indicating that NUDT16 PARylation may lead to dissociation of NUDT16 from PARP1 following DNA damage. Furthermore, the interaction of MBP-PARP1 with a PARylation deficient mutant (DDEEE5A) of NUDT16 resulted in no change in the interaction in the absence or presence of IR (Figure 8K). Thus, these data indicate that NUDT16 PARylation plays an important role in the IR-induced dissociation of PARP1 and NUDT16.

PARP1 has a modular structure with six domains (Supplemental Figure S7F, S7H): three zinc-finger DNA-binding domains (ZnF1, ZnF2, and ZnF3) in the N-terminus, a BRCA1 C-terminus (BRCT) domain, a Trp-Gly-Arg (WGR) domain, and the C-terminal catalytic domain (CAT). To define the binding regions between PARP1 and NUDT16, we performed co-immunoprecipitation (Co-IP) experiments. As shown in Supplemental Figure S7G, the N-terminal ZnF domain interacts with PARP1. Furthermore, our Co-IP experiments demonstrated that loss of ZnF3 of PAPR1 decreased its interaction with NUDT16 (Supplemental Figure S7I), indicating that ZnF3 of PARP1 is critical for its binding to NUDT16. Next, we sought to define the PARP1 binding region on NUDT16, thus we constructed several deletion mutations of NUDT16 (Supplemental Figure S7J). Our pulldown experiments demonstrated that deletion of the Nudix motif of NUDT16 drastically decreased the PARP1–NUDT16 interaction (Supplemental Figure S7K). These findings indicate that the PARP1–NUDT16 interaction is mediated by the ZnF3 domain of PARP1 and the Nudix motif of NUDT16.

## Discussion

As a member of Nudix hydrolase family, NUDT16 was original characterized as being able to catabolize nucleoside triphosphates and cap mRNAs (17,18) and has recently been shown to have hydrolase activities that remove protein ADP-ribosylation (19). More recently, we demonstrated that NUDT16’s catalytic hydrolase activity is required for removing PARylation chains from 53BP1, which regulates 53BP1 protein stability and its function in cell survival, providing yet another mechanism for 53BP1 regulation (20). Here, we began investigating whether there are other unidentified substrates of NUDT16 hydrolase activity.

NUDT16 regulates 53BP1 protein stability (20), highlighting the possible participation of NUDT16 in the DNA repair process. Here, we report multiple lines of evidence that NUDT16 is involved in the HR repair pathway. First, NUDT16 depletion reduces the HR repair capacity. Second, NUDT16 depletion decreases DNA end resection, which is the key step in the HR repair pathway. Third, loss of NUDT16 decreases CtIP recruitment to DSBs and the protein levels of CtIP, which is the key protein for initiating DNA end resection in the HR repair pathway. Sufficient end resection is necessary for RPA2 binding to ssDNA, which is essential for HR repair. Additionally, NUDT16 loss leads to a reduction of RPA2 phosphorylation, and this reduction is rescued by overexpression of NUDT16. Lastly, NUDT16 interacts with CtIP, which is mediated by His24, Arg50 and Asp52 residues of NUDT16 and Arg324, Ser326 and Ser327 residues of CtIP.

NUDT16 loss decreases CtIP protein levels, which may be due to CtIP degradation through the ubiquitin-proteasome pathway. Recent studies demonstrate that ADP-ribosylation can signal for ubiquitination and promote the degradation of ADP-ribosylated proteins (36–39). Indeed, our *in vitro* PARylation assays provide evidence that CtIP is ADP-ribosylated. Moreover, NUDT16 removes the PAR chains of CtIP. Therefore, ADP-ribosylation of CtIP is increased in NUDT16 KO cells. Additionally, PARPi treatment led to decreased PARylation and ubiquitination of CtIP, suggesting that ADP-ribosylated CtIP mediates its own ubiquitination and degradation. We further identified several ADP-ribosylation sites on CtIP that are critical for CtIP recruitment to DSBs and DNA end resection-initiated HR repair.

The E3 ligase RNF146 has been well described in ADP-ribosylation-dependent ubiquitination (40–42). We showed that ADP-ribosylated CtIP is targeted by RNF146, leading to CtIP ubiquitination and degradation. Both the WWE domain and RING domain of RNF146 are required for RNF146-mediated CtIP ubiquitination and degradation. However, RNF146 failed to facilitate the degradation of the RSS3A or SKEE4A mutant of CtIP, further supporting that RNF146-mediated CtIP ADP-ribosylation-dependent ubiquitination and degradation. Finally, NUDT16 loss promotes ADP-ribosylation of CtIP, which in turn leads to its ubiquitination and degradation by RNF146. Taken together, RNF146 is a *bona fide* E3 ligase for CtIP ADP-ribosylation-dependent ubiquitination. An important question remains whether there are other unidentified substrate proteins for NUDT16 hydrolase activity. If RNF146 is not the E3 ligase, the DELTEX (DTX) family of RING E3 ligases, in particular DTX2 and DTX3L, show a structural connection in the crosstalk between ADP-ribosylation and ubiquitination (50,51). We will test these possibilities by determining whether DTX2 or DTX3L is the E3 ligase for PARylation-dependent ubiquitination on other substrates.

It remains unclear how the enzymatic activity of NUDT16 is stimulated in response to DNA damage. The simplest mechanism is that PTMs, e.g. phosphorylation, ubiquitination, or poly (ADP-ribosyl)ation are induced by DNA damage. In this study, NUDT16 is ADP-ribosylated, which is important for CtIP protein stability, CtIP recruitment to DSBs, and for its hydrolase activity, HR repair function. In addition, the NUDT16-PARP1 interaction is decreased following IR-induced DNA damage. PARPi treatment prevents the dissociation of NUDT16 from PARP1, and the PARylation deficient mutant of NUDT16 does not alter the NUDT16-PARP1 interaction following DNA damage, suggesting that NUDT16 PARylation plays critical roles in IR-induced dissociation of NUDT16 from PARP1. NUDT16 may have a general role in poly (ADP-ribosyl)ation turnover at sites of DNA breaks and, therefore, influences the dynamic regulation of stepwise DNA repair processes. Whether or not PAR signals, which normally turn over rapidly at DSB sites, are delayed in cells expressing the catalytic inactive mutant or PARylation deficient mutant of NUDT16 remains to be determined.

Our current working model (Figure 8L) is that CtIP PARylation and de-PARylation are tightly controlled by NUDT16 to maintain a balance under physiological conditions. ADP-ribosylation is a previously unknown post-translational modification of CtIP. The ADP-ribosylated CtIP is targeted by a PAR-binding E3 ubiquitin ligase, RNF146, leading to CtIP ubiquitination and degradation. NUDT16 has hydrolyzing activity that cleaves ADP-ribosylation of CtIP and inhibits CtIP ubiquitination and degradation, stabilizing CtIP. In the presence of DNA damage, CtIP PARylation promotes CtIP recruitment to DSBs. NUDT16 PARylation leads to dissociation of NUDT16 from PARP1 following DNA damage, which may allow PARylated NUDT16 with hydrolase activity to remove ADP-ribosylation of CtIP, which ensures timely CtIP stability, and thus processes its DSB repair. We will further discover the underlying mechanism in our future studies.

We analyzed TCGA data and found that NUDT16 was expressed at lower levels in most pan-cancer tissues compared to normal tissues (Supplemental Figure S8A). Moreover, patient survival was in general longer with higher expression of NUDT16 (Supplemental Figure S8B). Interestingly, bioinformatics analysis of whole-exome sequencing data of cancer samples also helped us identify 60 missense mutations using Cancer-COSMIC and *cBioPortal*. Two mutants (E76K, E80K) are located in the core of the Nudix motif [R-E-x (2)-E-E], which is required for its hydrolase activity (19,20). The bioinformatics analysis further supports that a greater understanding of the underlying DNA repair pathways for NUDT16 may have a significant impact on the development of cancer.

## Supplementary Material

gkae064_Supplemental_File

## Data Availability

The data underlying this article are available in the article and in its online supplementary material.
