# Supplementary material for: NUDT16 regulates CtIP PARylation to dictate homologous recombination repair
Source: Nucleic Acids Res. 2024 Feb 7;52(7):3761–77. doi: 10.1093/nar/gkae064 (PMC11039996; doi:10.1093/nar/gkae064)
Supplement: gkae064_Supplemental_File [file gkae064_supplemental_file.pdf]

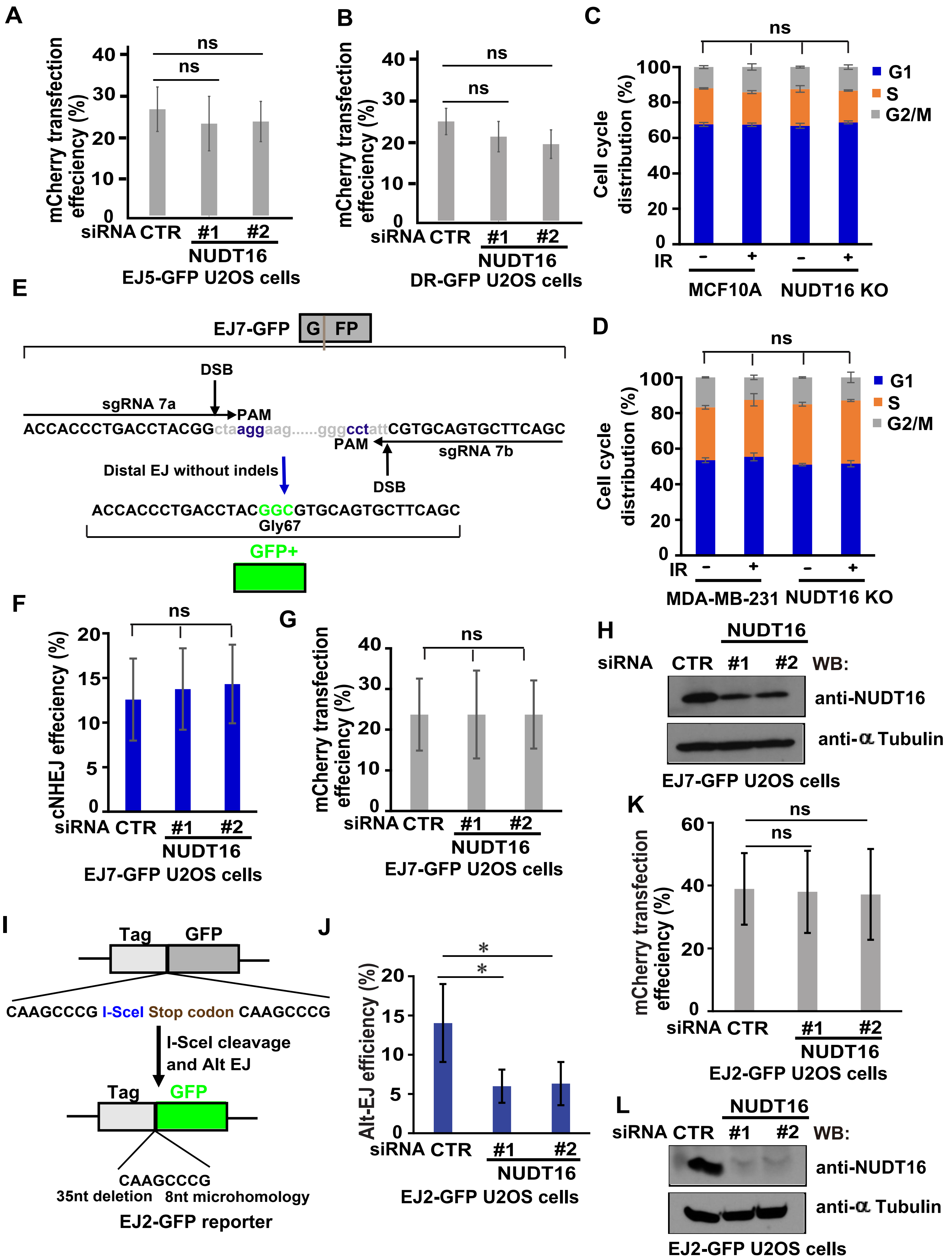
A
mCherry transfection efeciency (%)
ns
ns
siRNA CTR #1 #2
NUDT16
EJ5-GFP U2OS cells
B
mCherry transfection efeciency (%)
ns
ns
siRNA CTR #1 #2
NUDT16
DR-GFP U2OS cells
C
ns
Cell cycle distribution (%)
G1
S
G2/M
IR - + - +
MCF10A NUDT16 KO
D
ns
Cell cycle distribution (%)
G1
S
G2/M
IR - + - +
MDA-MB-231 NUDT16 KO
E
EJ7-GFP
G FP
DSB
sgRNA 7a
PAM
ACCACCCTGACCTACGGctaaggaag......gggcctattCGTGCAGTGCTTCAGC
PAM
sgRNA 7b
Distal EJ without indels
DSB
ACCACCCTGACCTACGGCGTGCAGTGCTTCAGC
Gly67
GFP+
F
ns
cNHEJ efeciency (%)
siRNA CTR #1 #2
NUDT16
EJ7-GFP U2OS cells
G
ns
mCherry transfection efeciency (%)
siRNA CTR #1 #2
NUDT16
EJ7-GFP U2OS cells
H
NUDT16
siRNA CTR #1 #2 WB:
anti-NUDT16
anti-α Tubulin
EJ7-GFP U2OS cells
I
Tag GFP
CAAGCCCG I-SceI Stop codon CAAGCCCG
I-SceI cleavage and Alt EJ
Tag GFP
CAAGCCCG
35nt deletion 8nt microhomology
EJ2-GFP reporter
J
*
*
Alt-EJ efficiency (%)
siRNA CTR #1 #2
NUDT16
EJ2-GFP U2OS cells
K
ns
ns
mCherry transfection efeciency (%)
siRNA CTR #1 #2
NUDT16
EJ2-GFP U2OS cells
L
NUDT16
siRNA CTR #1 #2 WB:
anti-NUDT16
anti-α Tubulin
EJ2-GFP U2OS cells

Zhang_Supplemental Figure S2

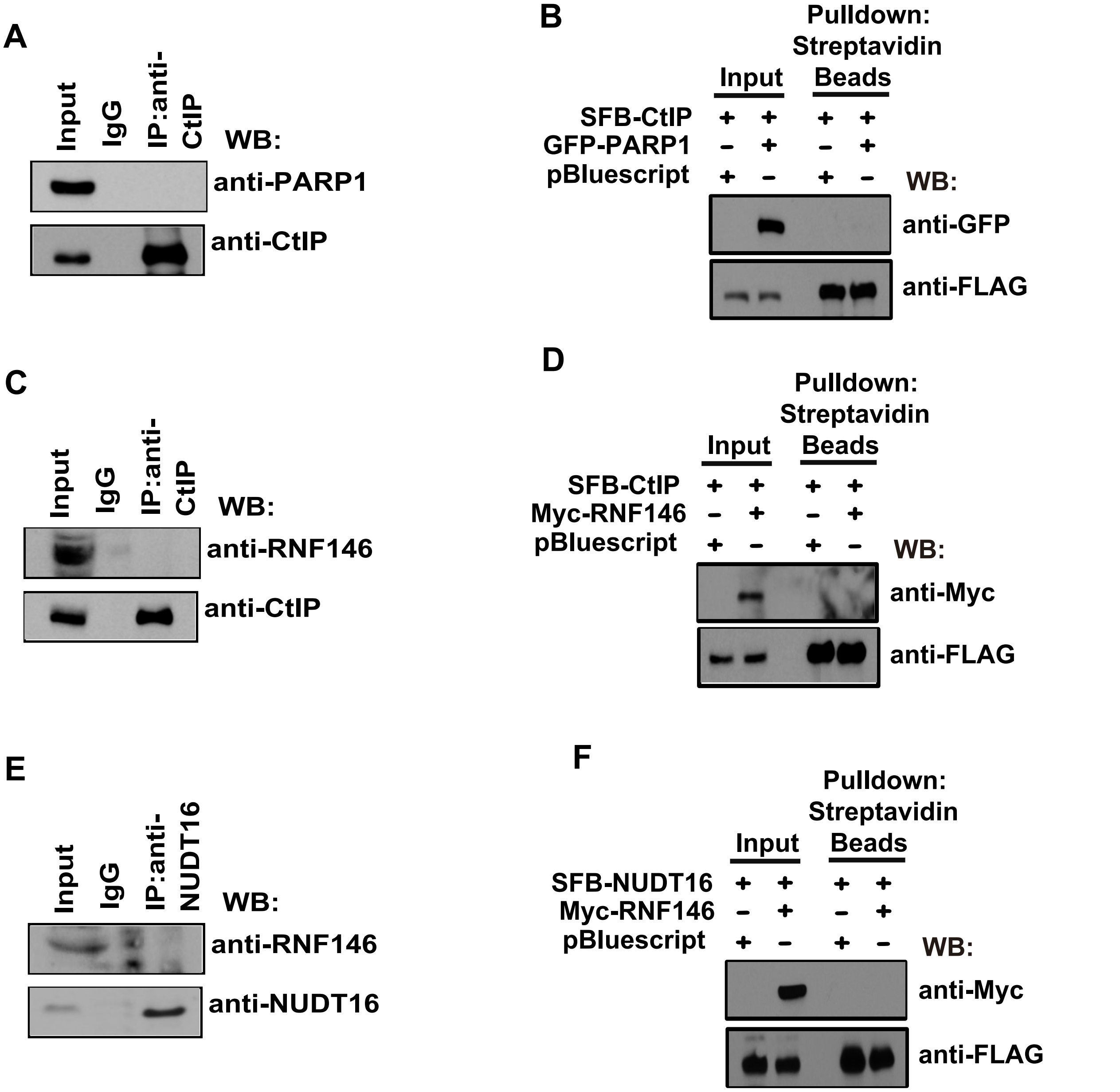

Zhang_Supplemental Figure S3

A

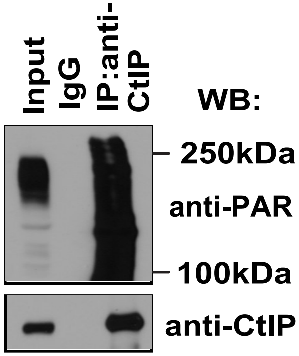

B

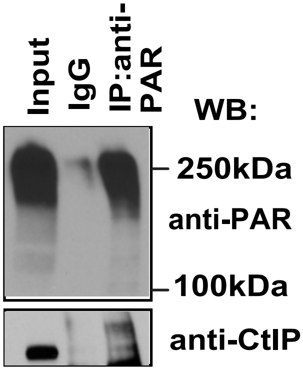

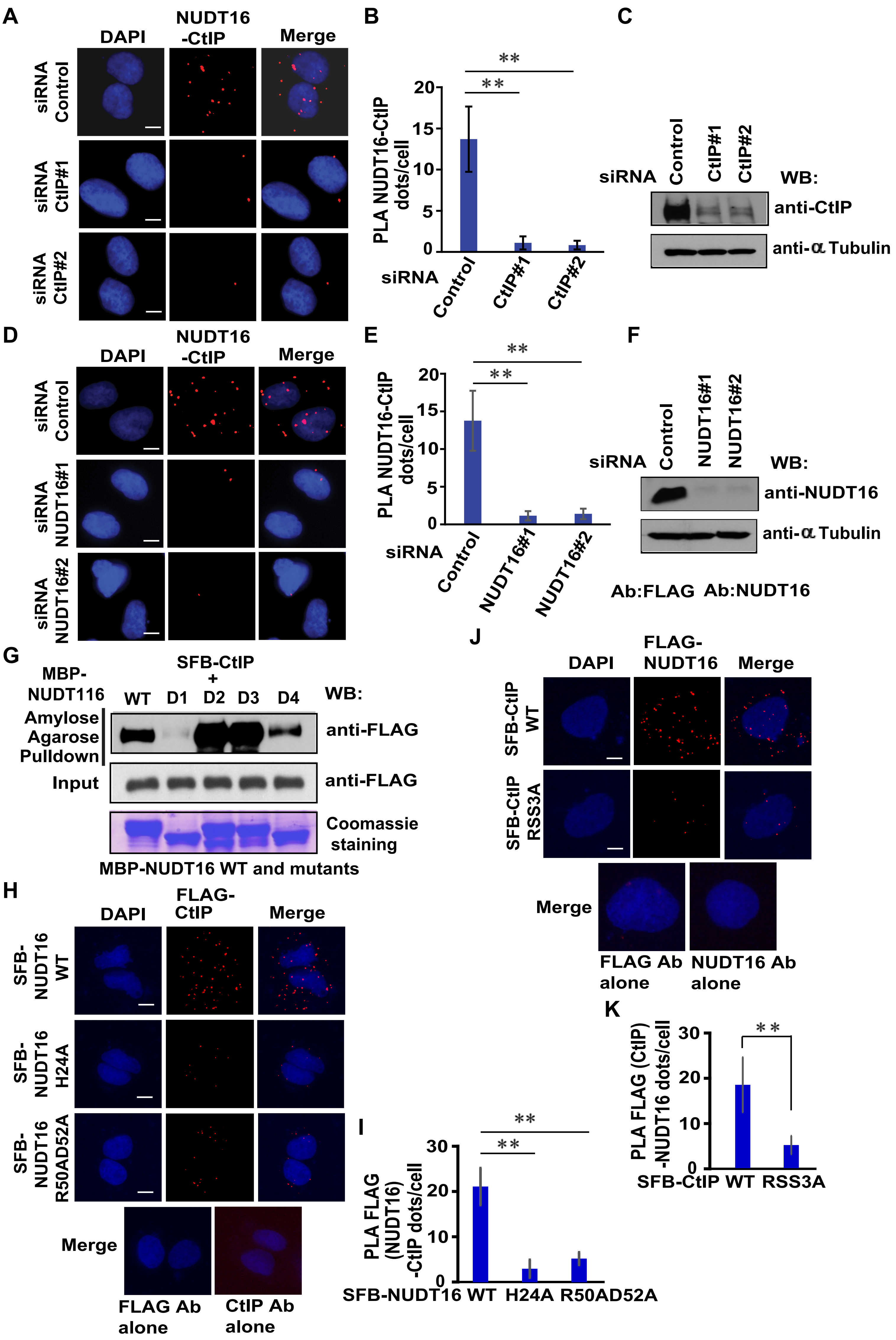
A
DAPI
NUDT16 -CtIP
Merge
siRNA Control
siRNA CtIP#1
siRNA CtIP#2
B
PLA NUDT16-CtIP dots/cell
siRNA
Control
CtIP#1
CtIP#2
C
siRNA
Control
CtIP#1
CtIP#2
WB:
anti-CtIP
anti-α Tubulin
D
DAPI
NUDT16 -CtIP
Merge
siRNA Control
siRNA NUDT16#1
siRNA NUDT16#2
E
PLA NUDT16-CtIP dots/cell
siRNA
Control
NUDT16#1
NUDT16#2
F
siRNA
Control
NUDT16#1
NUDT16#2
WB:
anti-NUDT16
anti-α Tubulin
G
MBP-NUDT116
SFB-CtIP +
WT D1 D2 D3 D4
WB:
Amylose Agarose Pulldown
anti-FLAG
Input
anti-FLAG
Coomassie staining
MBP-NUDT16 WT and mutants
H
DAPI
FLAG-CtIP
Merge
SFB-NUDT16 WT
SFB-NUDT16 H24A
SFB-NUDT16 R50AD52A
Merge
FLAG Ab alone
CtIP Ab alone
I
PLA FLAG (NUDT16) -CtIP dots/cell
SFB-NUDT16 WT H24A R50AD52A
J
Ab:FLAG Ab:NUDT16
DAPI
FLAG-NUDT16
Merge
SFB-CtIP WT
SFB-CtIP RSS3A
Merge
FLAG Ab alone
NUDT16 Ab alone
K
PLA FLAG (CtIP) -NUDT16 dots/cell
SFB-CtIP WT RSS3A

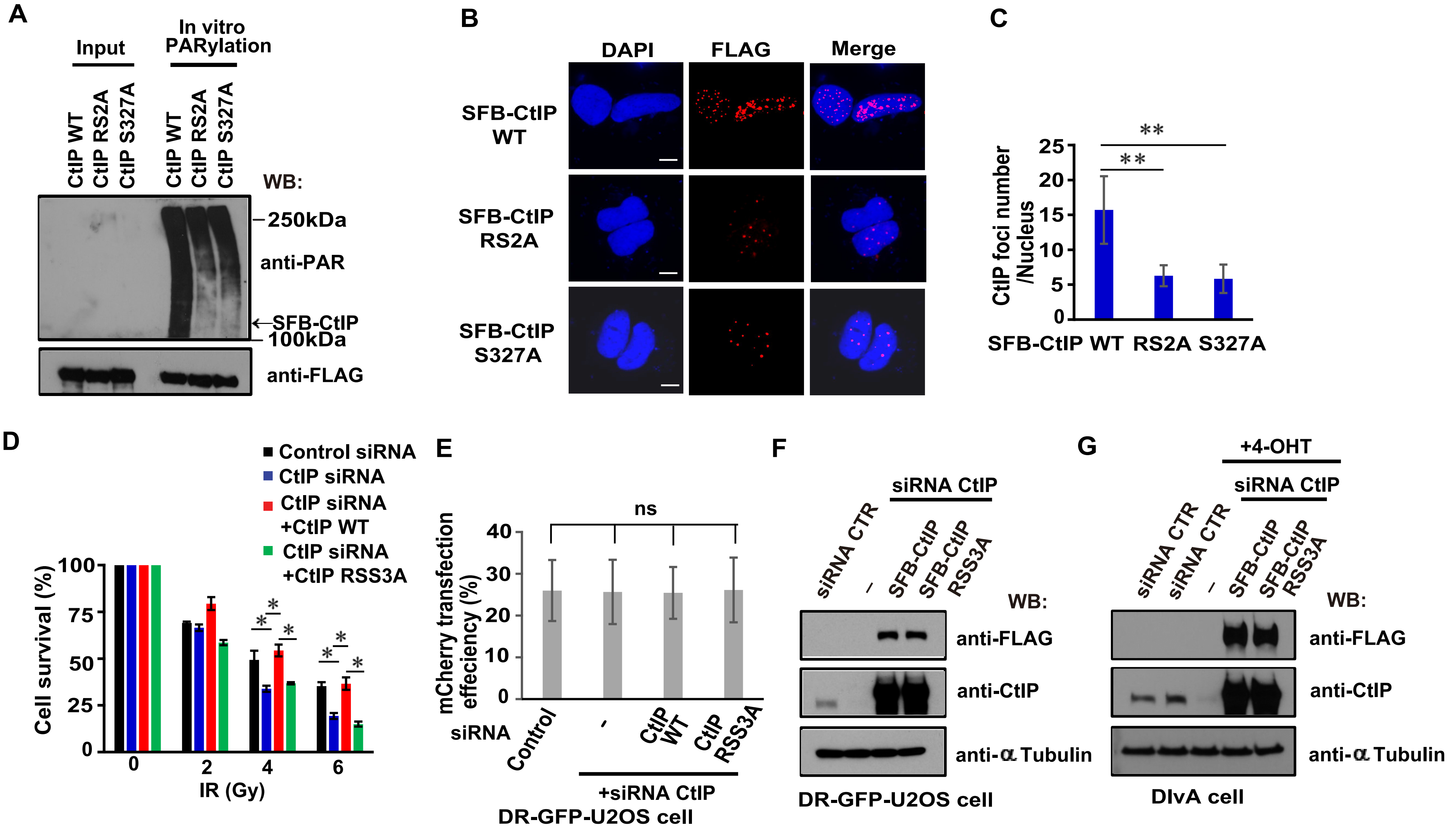
A
Input
In vitro PARylation
CtIP WT
CtIP RS2A
CtIP S327A
WB:
250kDa
anti-PAR
SFB-CtIP
100kDa
anti-FLAG
B
DAPI
FLAG
Merge
SFB-CtIP WT
SFB-CtIP RS2A
SFB-CtIP S327A
C
CtIP foci number /Nucleus
SFB-CtIP WT RS2A S327A
D
Control siRNA
CtIP siRNA
CtIP siRNA +CtIP WT
CtIP siRNA +CtIP RSS3A
Cell survival (%)
IR (Gy)
E
ns
mCherry transfection effeciency (%)
siRNA
Control
CtIP WT
CtIP RSS3A
+siRNA CtIP
DR-GFP-U2OS cell
F
siRNA CtIP
siRNA CTR
SFB-CtIP
SFB-CtIP RSS3A
WB:
anti-FLAG
anti-CtIP
anti-α Tubulin
DR-GFP-U2OS cell
G
+4-OHT
siRNA CtIP
siRNA CTR
siRNA CTR
SFB-CtIP
SFB-CtIP RSS3A
WB:
anti-FLAG
anti-CtIP
anti-α Tubulin
DIvA cell

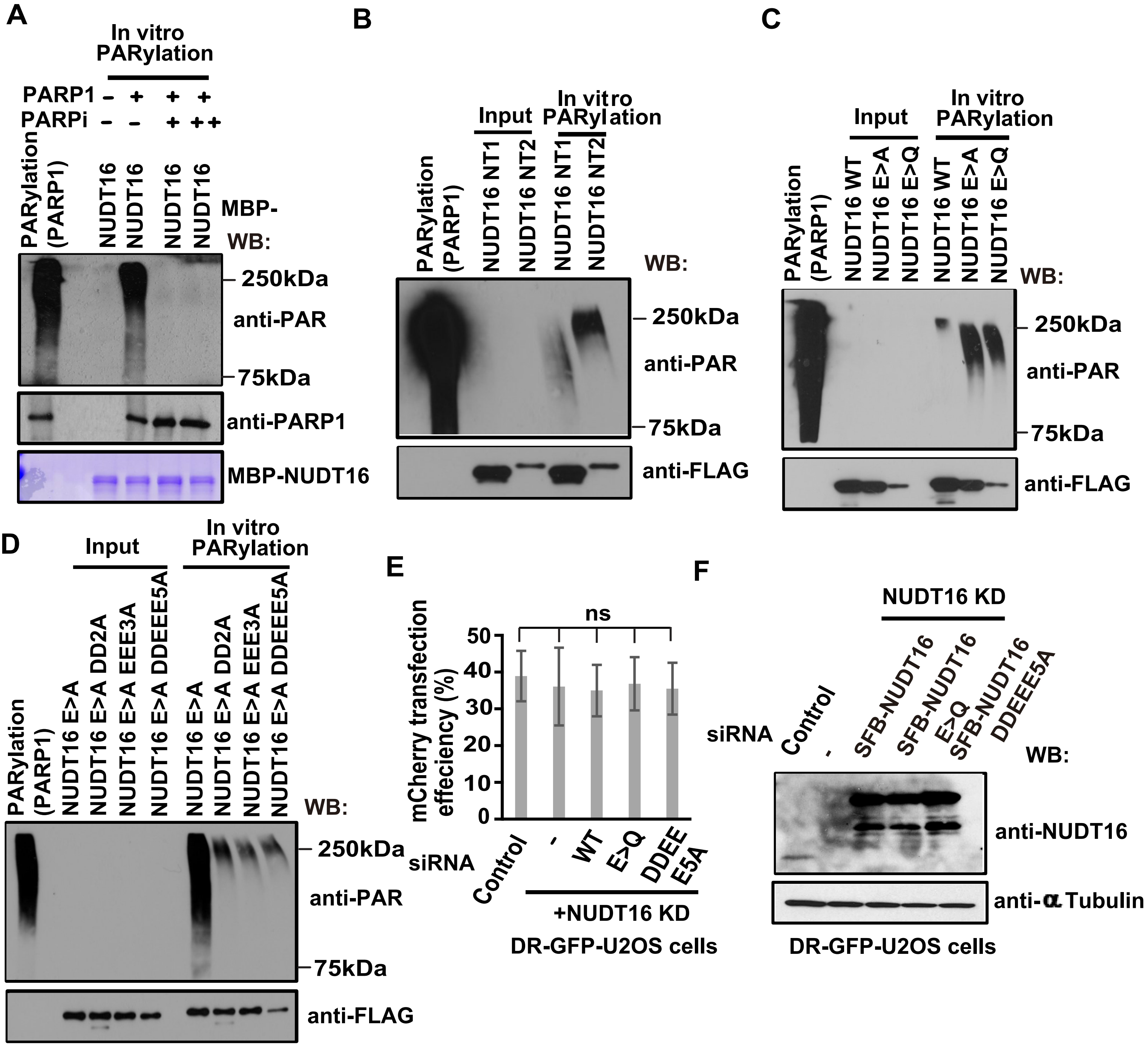
A
In vitro PARylation
PARP1 - + + +
PARPi - - + ++
PARylation (PARP1)
NUDT16
NUDT16
NUDT16
NUDT16
MBP-
WB:
250kDa
anti-PAR
75kDa
anti-PARP1
MBP-NUDT16
B
Input
In vitro PARylation
PARylation (PARP1)
NUDT16 NT1
NUDT16 NT2
NUDT16 NT1
NUDT16 NT2
WB:
250kDa
anti-PAR
75kDa
anti-FLAG
C
Input
In vitro PARylation
PARylation (PARP1)
NUDT16 WT
NUDT16 E>A
NUDT16 E>Q
NUDT16 WT
NUDT16 E>A
NUDT16 E>Q
WB:
250kDa
anti-PAR
75kDa
anti-FLAG
D
Input
In vitro PARylation
PARylation (PARP1)
NUDT16 E>A
NUDT16 E>A DD2A
NUDT16 E>A EEE3A
NUDT16 E>A DDEEE5A
NUDT16 E>A
NUDT16 E>A DD2A
NUDT16 E>A EEE3A
NUDT16 E>A DDEEE5A
WB:
250kDa
anti-PAR
75kDa
anti-FLAG
E
mCherry transfection effeciency (%)
ns
50
40
30
20
10
0
siRNA
Control
-
WT
E>Q
DDEE E5A
+NUDT16 KD
DR-GFP-U2OS cells
F
NUDT16 KD
siRNA
Control
-
SFB-NUDT16
SFB-NUDT16 E>Q
SFB-NUDT16 DDEEE5A
WB:
anti-NUDT16
anti-αTubulin
DR-GFP-U2OS cells

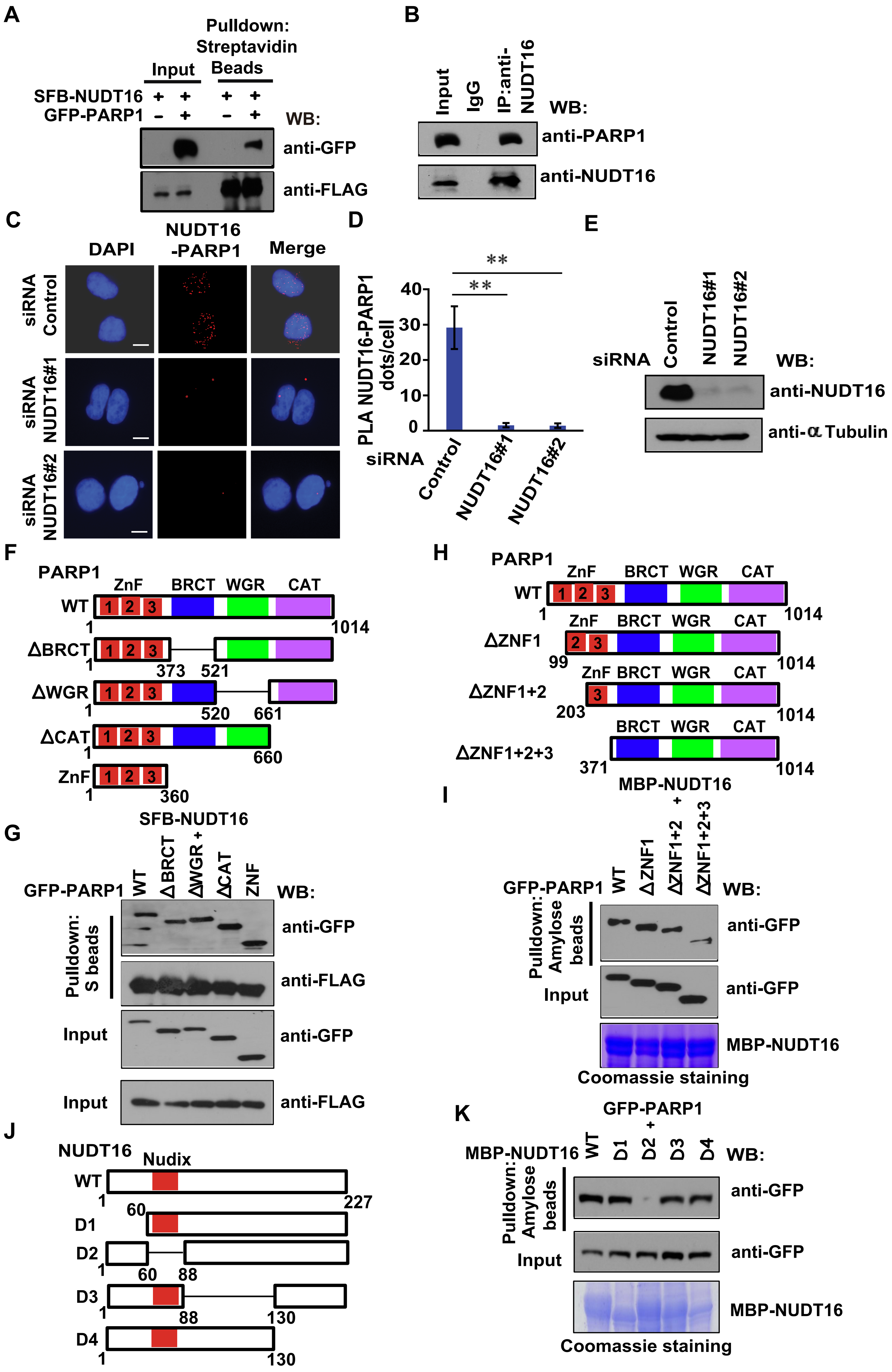
A
Pulldown: Streptavidin Beads
Input
SFB-NUDT16 + + + +
GFP-PARP1 – + – +
WB:
anti-GFP
anti-FLAG
B
Input
IgG
IP:anti-NUDT16
WB:
anti-PARP1
anti-NUDT16
C
DAPI
NUDT16 -PARP1
Merge
siRNA Control
siRNA NUDT16#1
siRNA NUDT16#2
D
PLA NUDT16-PARP1 dots/cell
40
30
20
10
0
**
**
siRNA
Control
NUDT16#1
NUDT16#2
E
siRNA
Control
NUDT16#1
NUDT16#2
WB:
anti-NUDT16
anti-α Tubulin
F
PARP1
ZnF
BRCT
WGR
CAT
WT
1
1014
ΔBRCT
373
521
ΔWGR
520
661
ΔCAT
660
ZnF
360
H
PARP1
ZnF
BRCT
WGR
CAT
WT
1
1014
ΔZNF1
99
ΔZNF1+2
203
ΔZNF1+2+3
371
G
SFB-NUDT16
GFP-PARP1
WT
ΔBRCT
ΔWGR +
ΔCAT
ZNF
WB:
Pulldown: S beads
anti-GFP
anti-FLAG
Input
anti-GFP
Input
anti-FLAG
I
MBP-NUDT16 +
GFP-PARP1
WT
ΔZNF1
ΔZNF1+2
ΔZNF1+2+3
WB:
Pulldown: Amylose beads
anti-GFP
Input
anti-GFP
MBP-NUDT16
Coomassie staining
J
NUDT16
Nudix
WT
1
227
D1
60
D2
60
88
D3
88
130
D4
130
K
GFP-PARP1 +
MBP-NUDT16
WT
D1
D2
D3
D4
WB:
Pulldown: Amylose beads
anti-GFP
Input
anti-GFP
MBP-NUDT16
Coomassie staining

A

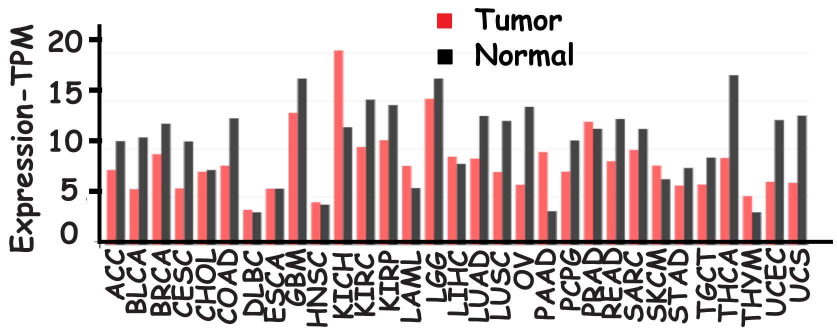
Tumor
Normal
Expression-TPM
20
15
10
5
0
ACC
BLCA
BRCA
CESC
CHOL
COAD
DLBC
ESCA
GBM
HNSC
KICH
KIRC
KIRP
LAML
LGG
LIHC
LUAD
LUSC
OV
PAAD
PCPG
PRAD
READ
SARC
SKCM
STAD
TGCT
THCA
THYM
UCEC
UCS

B

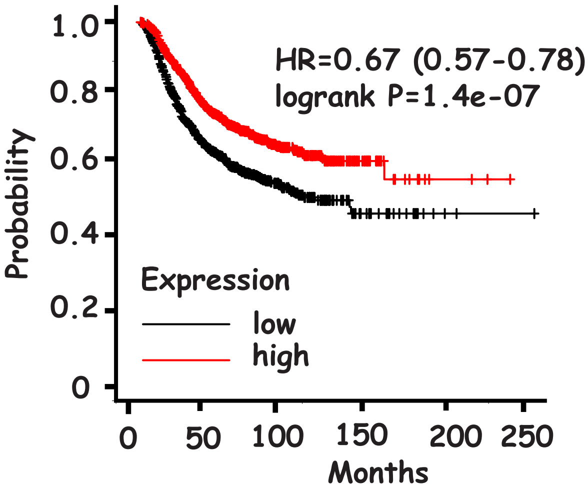
HR=0.67 (0.57-0.78)
logrank P=1.4e-07
Probability
1.0
0.8
0.6
0.4
0.2
0
Expression
low
high
0
50
100
150
200
250
Months

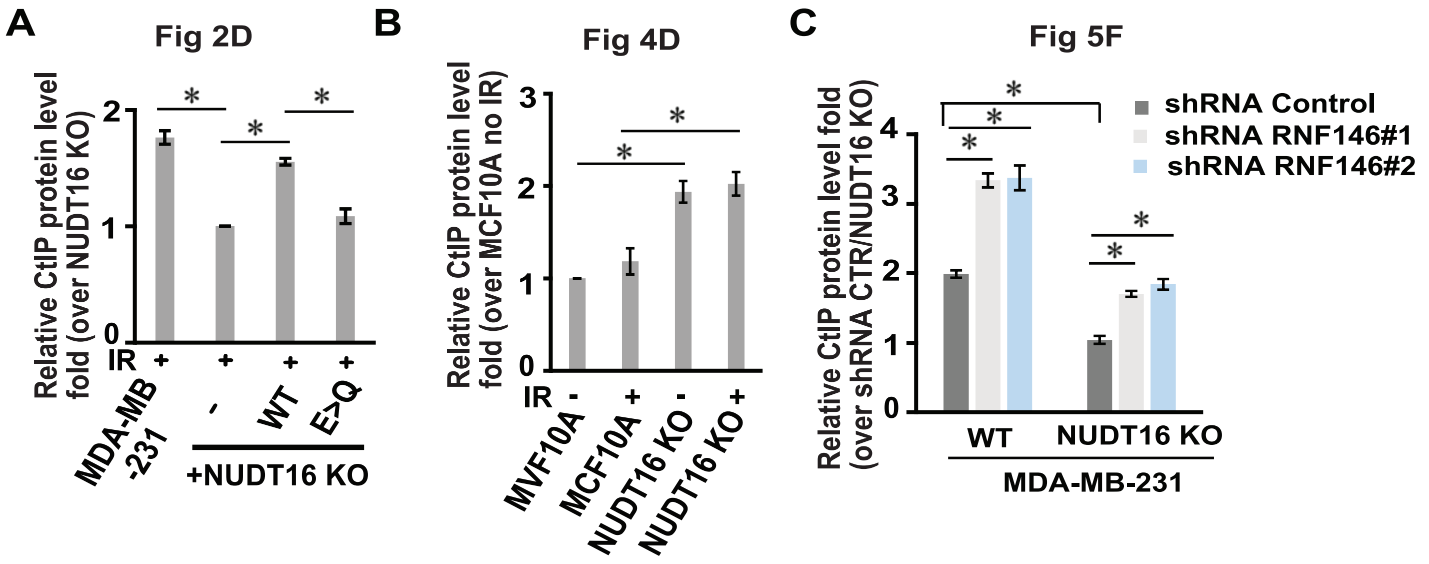
A
Fig 2D
Relative CtIP protein level fold (over NUDT16 KO)
2
1
0
IR + + + +
MDA-MB -231
-
WT
E>Q
+NUDT16 KO
B
Fig 4D
Relative CtIP protein level fold (over MCF10A no IR)
3
2
1
0
IR - + - +
MVF10A
MCF10A
NUDT16 KO
NUDT16 KO
C
Fig 5F
Relative CtIP protein level fold (over shRNA CTR/NUDT16 KO)
4
3
2
1
0
shRNA Control
shRNA RNF146#1
shRNA RNF146#2
WT
NUDT16 KO
MDA-MB-231

Supplemental Figure legends

Supplemental Figure S1. NUDT16 regulates DSB repair. (A) NUDT16 participates in NHEJ repair. 24 h after transfection with NUDT16 siRNA, EJ5-GFP U2OS cells were transfected with an I-SceI plasmid and pcDNA3.1-mCherry at a 9:1 ratio for 48 h. The mCherry$^+$ population was quantitated. Plasmids expressing mCherry were used to determine transfection efficiency. Data are represented as the mean ± S.E (n=3). ns, not significant. **(B)** NUDT16 participates in HR repair. 24 h after transfection with NUDT16 siRNA, DR-GFP U2OS cells were transfected with an I-SceI plasmid and pcDNA3.1-mCherry at a 9:1 ratio for 48 h. The mCherry$^+$ population was quantitated. Plasmids expressing mCherry were used to determine transfection efficiency. Data are represented as the mean ± S.E (n=3). ns, not significant. (**C**) MCF10A or (**D**) MDA-MB-231 and their derivative NUDT16 KO cells were collected at 1h after IR (20 Gy). Propidium iodide (PI) was used to stain the DNA, and the DNA content was quantified by FACS to monitor the cell cycle distribution of these cells. **(E-H)** NUDT16 is not involved in classical NHEJ repair. (**E**) Schematic representation of the EJ7-GFP reporter used to analyze the repair of Cas9-induced DSBs by classical NHEJ. (**F**) 24 h after transfection with NUDT16 siRNA, EJ7-GFP U2OS cells were transfected with mCherry and two sgRNAs, 7a and 7b at a 1:9 ratio for another 48 h. The GFP$^+$ and mCherry$^+$ cell population was quantitated and the cNHEJ rate represented as GFP$^+$/mCherry$^+$. Data are represented as the mean ± S.E. (n = 3). Note: ns, not significant. (**G**) Plasmids expressing mCherry were used to determine transfection efficiency. Data are represented as the mean ± S.E (n=3). Note: ns, not significant. (**H**) Western blotting analysis was performed to verify efficient silencing of NUDT16 following siRNA transfection in EJ7-GFP U2OS cells. (**I-L**) NUDT16 participates in Alt-EJ repair. (**I**) Schematic representation of the EJ2-GFP reporter used to analyze the repair of *I-SceI*-induced DSBs by Alt-EJ repair. (**J**) 24 h after transfection with NUDT16 siRNA, EJ2-GFP U2OS cells were transfected with an I-SceI plasmid and pcDNA3.1-mCherry at a 9:1 ratio for 48 h. The GFP$^+$ and mCherry$^+$ population was quantitated, and the Alt-EJ rate represented as GFP$^+$/mCherry$^+$. Data are represented as the mean ± S.E. (n = 3). *, $p < 0.05$. (**K**) Plasmids expressing mCherry were used to determine transfection efficiency. Data are represented as the mean ± S.E (n=3). Note: ns, not significant. (**L**) Western blotting analysis was performed to verify efficient silencing of NUDT16 following siRNA transfection in EJ2-GFP U2OS cells.

Supplemental Figure S2. CtIP does not interact with PARP1 and RNF146. (A) Endogenous CtIP does not interact with PARP1. HEK293T cell lysates were prepared and immunoprecipitated with CtIP antibody followed by immunoblotting with the PARP1 antibody. **(B)** CtIP does not

interact with PARP1 by Co-IP experiment. HEK293T cells were transfected with plasmids encoding SFB-tagged CtIP together with pBluescript or GFP-tagged PARP1. Co-IP reactions were performed with streptavidin beads and subjected to western blotting with the indicated antibodies. **(C)** Endogenous CtIP does not interact with RNF146. HEK293T cell lysates were prepared and immunoprecipitated with CtIP antibody followed by immunoblotting with the RNF146 antibody. **(D)** CtIP does not interact with RNF146 by Co-IP experiment. HEK293T cells were transfected with plasmids encoding SFB-tagged CtIP together with pBluescript or Myc-tagged RNF146. Co-IP reactions were performed with streptavidin beads and subjected to western blotting with the indicated antibodies. **(E)** Endogenous NUDT16 does not interact with RNF146. HEK293T cell lysates were prepared and immunoprecipitated with NUDT16 antibody followed by immunoblotting with the RNF146 antibody. **(F)** NUDT16 does not interact with RNF146 by Co-IP experiment. HEK293T cells were transfected with plasmids encoding SFB-tagged NUDT16 together with pBluescript or Myc-tagged RNF146. Co-IP reactions were performed with streptavidin beads and subjected to western blotting with the indicated antibodies.

Supplemental Figure S3. CtIP is ADP-ribosylated *in vivo*. MCF10A cells were harvested, and the lysates were immunoprecipitated with CtIP antibody (**A**) or PAR antibody (**B**). IgG was used as negative control. Immunoprecipitates were blotted using the indicated antibodies.

Supplemental Figure S4. CtIP is a novel NUDT16-interacting protein. **(A-F)** PLA detection of the NUDT16-CtIP interaction. (**A, D**) U2OS cells were transfected with the control siRNA, NUDT16 siRNA, or CtIP siRNA and were subjected to PLA using the NUDT16 antibody and CtIP antibody, shown as distinct fluorescent dots. Scale bar, 10 μM. (**B, E**) PLA dots per cell were quantified in 50 cells for each experiment. Data are represented as the mean ± S.E. (n = 3). **, $p < 0.01$. (**C, F**) Western blotting was performed to assess siRNA mediated knockdown efficiency in cells. **(G)** The N terminus of NUDT16 is critical for the NUDT16-CtIP interaction. Beads coated with bacterially expressed and purified MBP-NUDT16 WT, or deletion mutants' fusion proteins were incubated, respectively, with cell lysates containing exogenously expressed SFB-CtIP. Immunoblotting experiments were carried out using the indicated antibodies. (**H-I**) PLA detection of the interaction between SFB-NUDT16 and endogenous CtIP. (**H**) U2OS cells were transfected with SFB-NUDT16 WT, the H24A mutant, and the R50AD52A mutant. The PLA assay was performed using the CtIP antibody and FLAG antibody, shown as distinct fluorescent dots. negative controls used either one of the two antibodies. Scale bar, 10 μm. (**I**) Quantification of the results in (**H**). PLA dots per cell were quantified in 50 cells for each experiment. Data are represented as the mean ± S.E. (n = 3). **, $p < 0.01$. (**J-K**) PLA detection of the interaction

between SFB-CtIP and endogenous NUDT16. (**J**) U2OS cells were transfected with SFB-CtIP WT or the RSS3A mutant. The PLA assay was performed using the NUDT16 antibody and FLAG antibody, shown as distinct fluorescent dots. Negative controls used either one of the two antibodies. Scale bar, 10 µm. (**K**) Quantification of the results in (**J**). PLA dots per cell were quantified in 50 cells for each experiment. Data are represented as the mean ± S.E. (n = 3). **, $p < 0.01$.

Supplemental Figure S5. CtIP PARylation is involved in DNA damage response. **(A)** Identification of ADP-ribosylation sites on CtIP. *In vitro* PARylation assays were performed as described in **Fig 3A**. Note: RS2A: R324AS326A; **(B)** ADP-ribosylation of CtIP is required for CtIP localization at DSBs. U2OS cells were transfected with plasmids encoding WT, RS2A, or S327A mutant of SFB-CtIP, respectively, then followed by IR (10Gy). One hours later, the cells were fixed and immunostained with anti-FLAG antibody. (**C**) Quantification of the results in (**B**). The CtIP foci number were quantified in 50 cells for each experiment. Data are represented as the mean ± S.E. (n = 3). **, $p < 0.01$. (**D**) CtIP PARylation affects cellular sensitivity to IR. CtIP depleted U2OS cells were reconstituted with siRNA-resistant WT or RSS3A mutant of SFB-tagged CtIP. These cells were mock-treated or treated with different doses of IR. Colony formation was quantified relative to colonies formed in untreated cells from the same setting. Data are represented as the mean ± S.E. (n = 3). *, $p < 0.05$. (**E-F**) Defect in CtIP PARylation impairs HR repair. DR-GFP-U2OS cells were reconstituted with siRNA-resistant WT or RSS3A mutant of SFB-tagged CtIP. After CtIP depletion using siRNA for 24 h, these cells were transfected with I-SceI plasmid and pcDNA3.1-mCherry at 9:1 ratio for another 48 h. (**E**) Plasmids expressing mCherry were used to determine transfection efficiency. Data are represented as the mean ± S.E (n=3). Note: ns, not significant. (**F**) The indicated cell lines were collected and were immunoblotted with the indicated antibodies. (**G**) CtIP ADP-ribosylation affects DNA end resection activity. DIvA cells were reconstituted with siRNA-resistant WT or RSS3A mutant of SFB-tagged CtIP. These cells pretreated with CtIP siRNA were incubated with 4-OHT for 4 h. The indicated cell lines were collected and were immunoblotted with the indicated antibodies.

Supplemental Figure S6. NUDT16 is ADP-ribosylaed. (A) NUDT16 is ADP-ribosylated by PARP1. *in vitro* PARylation assays were performed in a reaction mix consisting of bacterially expressed NUDT16 proteins, with or without PARP1 enzyme, 1x reaction buffer, 20 µM NAD+, and 1x active DNA for 30 min at room temperature. PARP1 containing reactions were treated with or without PARPi (20µM, 40µM). PARP1 enzyme serves as a positive control in a reaction mix without NUDT16 proteins. The samples were immunoblotted with the indicated antibodies. **(B)**

Map of the ADP-ribosylation regions on NUDT16. *In vitro* PARylation assays were performed as described in **Fig 3A**. Note: NT1: 1-62aa; NT2: 63-130aa. (**C**) Increased PARylation signal is detected in NUDT16 catalytic inactive mutants. *In vitro* PARylation assays were performed as described in **Fig 3A**. Note: E>Q: E76QE79QE80Q; E>A: E76AE79AE80A. (**D**) Verification of ADP-ribosylation sites on NUDT16. The *in vitro* PARylation assays were performed as described in **Fig 3A**. Note: E>A DD2A: E76AE79AE80A/D63AD66A; E>A EEE3A: E76AE79AE80A/ E118AE119AE124A; E>A DDEEE5A: E76AE79AE80A/D63AD66AE118AE119AE124A. (**E-F**) NUDT16 PARylation participates in HR repair. (**E**) Plasmids expressing mCherry were used to determine transfection efficiency. Data are represented as the mean ± S.E (n=3). Note: ns, not significant. (**F**) Western blotting analysis was performed to verify efficient silencing of NUDT16 following siRNA transfection in DR-GFP U2OS cells.

Supplemental Figure S7. NUDT16 interacts with PARP1. **(A)** The NUDT16-PARP1 interaction is not mediated by DNA. HEK293T cells were transfected with plasmids encoding SFB-tagged NUDT16 and GFP-tagged PARP1. Cells were lysed with NTEN buffer containing protease inhibitors, phosphatase inhibitors, 2 mM MgCl2 and Benzonase at 4°C for 30 min. Co-IP reactions were conducted with streptavidin beads and subjected to western blotting with the indicated antibodies. **(B)** The NUDT16-PARP1 interaction is not mediated by DNA. HEK293T cells were lysed with NTEN buffer containing protease inhibitors, phosphatase inhibitors, 2 mM MgCl2 and Benzonase at 4°C for 30 min. Cell lysates were prepared and immunoprecipitated with the NUDT16 antibody followed by immunoblotting with the indicated antibodies. **(C-E)** PLA detection of the NUDT16-PARP1 interaction. (**C**) U2OS cells were transfected with the control siRNA and NUDT16 siRNA and were subjected to PLA using the NUDT16 antibody and PARP1 antibody, shown as distinct fluorescent dots. Scale bar, 10 μM. (**D**) PLA dots per cell were quantified in 50 cells for each experiment. Data are represented as the mean ± S.E. (n = 3). **, $p < 0.01$. (**E**) Western blotting was performed to assess siRNA mediated knockdown efficiency in cells. (**F**) Schematic representation of wild-type (WT) and deletion mutants of PARP1 used in this study. (**G**) The ZnF domain of PARP1 is required for the NUDT16-PARP1 interaction. HEK293T cells were transfected with plasmids encoding SFB-NUDT16 along with plasmids encoding the WT or deletion mutants of GFP-PARP1. Co-immunoprecipitation reactions were conducted with S beads and subjected to western blotting with the indicated antibodies. (**H**) Schematic representation of WT and ZnF deletion mutants of PARP1 used in this study. (**I**) The ZnF3 domain of PARP1 is necessary for NUDT16-PARP1 interaction. Beads coated with bacterially expressed MBP-NUDT16 fusion protein were incubated with HEK293T cell lysates containing exogenously expressed WT or ZnF deletion mutants of GFP-PARP1. Immunoblotting experiments were carried

out with the indicated antibodies. (**J**) Schematic representation of WT and deletion mutants of NUDT16 used in this study. (**K**) The Nudix domain of NUDT16 is required for NUDT16-PARP1 interaction. Beads coated with bacterially expressed MBP-NUDT16 and its deletion mutants' fusion proteins were incubated with HEK293T cell lysates containing exogenously expressed GFP-PARP1, respectively. Immunoblotting experiments were carried out with the indicated antibodies.

Supplemental Figure S8. Pan-cancer analysis on NUDT16 gene using TCGA data. (A) Pan-cancer analysis on mRNA expression levels of NUDT16 in normal (black) versus tumor (red) patient TCGA samples. **(B)** Kaplan–Meier curves of overall survival (OS) in pan-cancer patients.

Supplemental Figure S9. Quantification of the western blot bands' relative CtIP expression in Figures using ImageJ. (**A**) Figure 2D. (**B**) Figure 4D. (**C**) Figure 5F.
